# Acute upper gastrointestinal bleeding in the UK: 2022 audit update

**DOI:** 10.1136/gutjnl-2025-335134

**Published:** 2025-11-19

**Authors:** Gaurav B Nigam, Kathryn Oakland, Sarah Hearnshaw, John Grasnt-Casey, Paul Davies, Paula Dhiman, Shane W Goodwin, Bhaskar Kumar, Elizabeth Ratcliffe, Joanna A Leithead, Raman Uberoi, Lise Estcourt, Vipul Jairath, Simon P L Travis, Mike F Murphy, Adrian Stanley, Andrew Douds, AUGIB audit 2022 collaborator, UK

**Affiliations:** 1 Translational Gastroenterology and Liver Unit, Oxford University Hospitals NHS Trust, Oxford, UK; 2 Digestive Diseases Department, HCA Healthcare UK, London, UK; 3 Newcastle Upon Tyne Hospitals NHS Foundation Trust, Newcastle upon Tyne, UK; 4 National Comparative Audit Office, NHS Blood and Transplant, Oxford, UK; 5 Center for Statistics in Medicine, University of Oxford Nuffield Department of Orthopaedics Rheumatology and Musculoskeletal Sciences, Oxford, UK; 6 NIHR Oxford Biomedical Research Centre, Oxford, UK; 7 Department of Medicine, Lawson Health Research Institute, London, Ontario, Canada; 8 Upper Gastrointestinal Surgery, Norfolk and Norwich University Hospitals NHS Foundation Trust, Norwich, UK; 9 Wrightington Wigan and Leigh NHS Foundation Trust, Wigan, UK; 10 Forth Valley Royal Hospital, Larbert, UK; 11 Department of Interventional Radiology, Oxford University Hospitals NHS Foundation Trust, Oxford, UK; 12 NHS Blood and Transplant, Oxford University Hospitals NHS Foundation Trust, Oxford, UK; 13 Department of Medicine, Division of Gastroenterology, Western University Schulich School of Medicine & Dentistry, London, Ontario, Canada; 14 Department of Epidemiology and Biostatistics, Western University, London, Ontario, Canada; 15 Kennedy Institute of Rheumatology, Nuffield Department of Orthopaedics, Rheumatology and Musculoskeletal Sciences and, Biomedical Research Centre, University of Oxford, Oxford, UK; 16 NHS Blood and Transplant; Department of Haematology, Oxford University Hospitals NHS Foundation Trust, Oxford, UK; 17 Radcliffe Department of Medicine, University of Oxford, Oxford, UK; 18 Gastroenterology, Glasgow Royal Infirmary, Glasgow, UK; 19 Gastroenterology, Norfolk and Norwich University Hospital NHS Trust, Norwich, UK

**Keywords:** GASTROINTESTINAL HAEMORRHAGE, ENDOSCOPY, GASTROINTESTINAL BLEEDING, AUDIT

## Abstract

**Background:**

Acute upper gastrointestinal bleeding (AUGIB) is a common medical emergency with evolving demographics and management strategies, particularly in medical/endoscopic therapy and transfusion strategies.

**Objective:**

To provide key data of the most recent 2022 UK audit and compare it with the preceding audit in 2007.

**Design:**

Prospective multicentre audit conducted from 3 May to 2 July 2022, including adults (≥16 years) with AUGIB across 147 UK hospitals (response rate 86% vs 84% in 2007). AUGIB was defined by clinical symptoms (haematemesis, haematochezia, coffee ground vomiting or melaena confirmed by medical personnel). Patients were followed until discharge, death or 28 days, with re-admissions during the study period counted as new episodes.

**Results:**

Among 5141 patients (59% male; median age 69), 15% had cirrhosis, 19% reported excess alcohol use, 7% used non-steroidal anti-inflammatory drugs (NSAIDs) and 46% were on antithrombotics. Most (77%) were new admissions, who were younger with fewer comorbidities, while the remainder bled during hospitalisation. Peptic ulcer disease accounted for 32% of cases, varices for 10% and no abnormality was found in 33%. Pre-endoscopic risk stratification was not performed in 42%.

Compared with 2007, patients in 2022 had higher comorbidity (67% vs 50%), more cirrhosis (15% vs 9%), greater anticoagulant use (31% vs 13%) and higher transfusion rates (50% vs 43%). In 2022, among early transfusions (pre-endoscopy or within first 24 hrs; 38%), 43% were given at haemoglobin (Hb)>70 g/L, with 24% classified as inappropriate due to haemodynamic stability. A signal of harm was observed: while inappropriate transfusion was not associated with rebleeding at either 70 or 80 g/L, at 80 g/L it was linked to higher adjusted mortality (adjusted OR (aOR) 1.60, 95% CI 1.00 to 2.56).

Inpatient endoscopy was more common (83% vs 74%), though endotherapy use remained modest (27% vs 23%). Salvage therapy rates were unchanged (3.3% vs 3.1%) but shifted from surgery to interventional radiology. Outcomes improved, with lower rebleeding (9.7% vs 13.3%), reduced in-hospital mortality (8.8% vs 10.0%) and shorter median stay (5 vs 6 days). In multivariate analysis, mortality was independently predicted by older age (≥80 years: aOR 2.32, 95% CI 1.64 to 3.30), shock (aOR 2.22, 95% CI 1.53 to 3.17) and comorbidity, while lower Hb at presentation increased risk (≤70 g/L: aOR 1.56, 95% CI 1.15 to 2.11). Anticoagulant use was associated with increased mortality (aOR 1.43, 95% CI 1.11 to 1.85), whereas NSAID use (aOR 0.49, 95% CI 0.25 to 0.96) and antiplatelet use (aOR 0.68, 95% CI 0.54 to 0.87) were associated with lower mortality.

**Conclusions:**

Despite a higher-risk case mix and incomplete adherence to guidelines (notably in transfusion thresholds and risk stratification), outcomes in AUGIB have improved. The observation of increased mortality with liberal transfusion above 80 g/L in stable patients reinforces the importance of restrictive transfusion practice. Quality improvement initiatives focused on risk stratification, endoscopic training and multidisciplinary care could further enhance outcomes in the UK and internationally.

**WHAT IS ALREADY KNOWN ON THIS TOPIC**

- Acute upper gastrointestinal bleeding is a common medical emergency with significant morbidity and mortality. The 2007 UK audit highlighted gaps in care, including overuse of liberal transfusion strategies and limited access to timely interventions.

**WHAT THIS STUDY ADDS**

- This audit of UK practice shows improved overall outcomes, with reduced rebleeding and mortality rates, increased use of endoscopic and radiological interventions and less reliance on surgery, despite an ageing population with more comorbidities and anticoagulant use. Patient risk stratification and transfusion strategies were still suboptimal.

**HOW THIS STUDY MIGHT AFFECT RESEARCH, PRACTICE OR POLICY**

- These findings reinforce the value of evidence-based care pathways but highlight variation in adherence to national guidelines for transfusion. Further studies that explore the impact of liberal transfusion on patient outcomes are needed to either influence changes in practice or challenge current guidelines. There is also a need for ongoing training in haemostatic endoscopic techniques, and equitable access to specialised care.

## Introduction

 Acute upper gastrointestinal bleeding (AUGIB) is a medical emergency, associated with significant morbidity and mortality worldwide.^1^ In the UK, it has an annual incidence of 134 per 100 000, equating to approximately 60 000 hospital admissions annually and accounting for 11% of red blood cell (RBC) transfusions in hospitals.^2 3^

The 2007 UK-wide AUGIB audit reported in-hospital mortality rates of 7% for new admissions and 26% for inpatients.^4^ This landmark audit informed national and international guidelines, including the 2012 National Institute for Health and Care Excellence guidelines and the 2015 National Confidential Enquiry into Patient Outcome and Death report on gastrointestinal bleeding (GIB), and helped shape clinical care protocols in AUGIB management.^4^,^8^ Notably, it also revealed that early RBC transfusion was associated with a twofold increase in rebleeding risk and a higher mortality rate, likely due to the liberal transfusion strategies employed at the time.^9^ These findings laid the groundwork for subsequent trials, including a UK cluster randomised trial, transfusion for AUGIB (TRIGGER) trial, comparing restrictive and liberal transfusion strategies.^10^,^12^

Over the past 15 years, advances in AUGIB management have transformed clinical practice.^13^ Restrictive transfusion strategies have been informed by pivotal trials in AUGIB.^10 12^ Similarly, increased access to 24/7 emergency endoscopy and interventional radiology (IR) may have facilitated timely, minimally invasive haemorrhage control, possibly reducing reliance on surgical interventions.^14^ These changes warrant an evaluation of their real-world impact on clinical management and patient outcomes. Simultaneously, the ageing patient population with increasing comorbidities and the widespread use of antithrombotics (antiplatelets and anticoagulants) has introduced new complexities, necessitating re-evaluation of clinical pathways and outcomes.

The 2022 UK-wide audit was conducted to provide an updated overview of AUGIB in the UK, reflecting current patient characteristics, management practices and outcomes across a broad range of hospitals. It also aimed to assess changes over time by comparing findings with the 2007 national audit to inform future service planning and care pathways, and to identify key pre-endoscopy factors associated with mortality.

## Methods

The 2022 UK-wide audit was a prospective, multicentre observational study conducted from 3 May to 2 July 2022, in collaboration with the British Society of Gastroenterology (BSG), National Health Service (NHS) Blood and Transplant, Royal College of Physicians, British Association for Study of Liver diseases, Scottish Society of Gastroenterology, Association of Upper Gastrointestinal Surgeons and British Society of Interventional Radiology. All NHS acute-admitting hospitals in the UK were invited to participate. The audit collected data on established methods of care, involved no interventions and excluded patient identifiers, making it exempt from NHS Research Ethics Committee review.^15^ Routinely available clinical information collected as part of standard care was used for the clinical audit. According to NHS guidelines, this process is exempt from requiring prior consent and this study follows similar methodology to previous UK-wide audits.^4 16 17^ A validation study linking the 2007 audit to Hospital Episode Statistics confirmed accurate case ascertainment using this approach.^18^ The project was locally registered at participating hospitals with local information governance procedures in place. The study protocol is available online: https://osf.io/zet8r/.^19^

### Case definitions

Patients aged ≥16 years with suspected or confirmed AUGIB presenting to emergency departments or developing AUGIB while hospitalised for another reason were eligible. Patients transferred from other hospitals for management of AUGIB were also included. Inclusion criteria, similar to those used in 2007, were based on clinical symptoms (eg, melaena, haematemesis, syncope, coffee-ground vomiting), not investigation findings or discharge diagnoses. Patients with symptomatic iron-deficiency anaemia without signs of AUGIB were excluded. Definitions are provided in box 1.

Box 1 **Definitions used in the study**

- Acute upper gastrointestinal bleeding (AUGIB): Haematemesis, the passage of melaena and/or firm clinical evidence supported by laboratory findings of acute blood loss originating from the upper gastrointestinal (UGI) tract. Cases of ‘coffee ground’ vomiting were to be included only if witnessed by medical or nursing staff. Patients presenting with iron deficiency anaemia without evidence of acute bleeding were excluded.
- Haematemesis: Vomiting of blood or blood clots.
- Melaena: The passage of dark, tarry stools as observed by medical or nursing staff or identified during rectal examination.
- Haematochezia: The passage of fresh or altered blood per rectum, typically indicative of brisk UGI bleeding when associated with haemodynamic instability or other evidence of acute blood loss.
- Alcohol excess: Consumption exceeding 14 units of alcohol per week.
- Alcohol use: Any documented history of regular alcohol intake.
- Shock: defined as systolic blood pressure <100 mm Hg and heart rate ≥100, based on observations recorded at the time of presentation.
- Higher level care: Refers to advanced clinical management provided in specialised settings, including:
  - Level II/high dependency unit: For patients requiring closer monitoring and support than available in a general ward, such as invasive monitoring or single-organ support.
  - Level III/intensive therapy unit: For patients needing full intensive care, including multiorgan support or mechanical ventilation.
- Rebleeding (further bleeding): Recurrent haematemesis, coffee ground vomiting or bloody nasogastric aspirate following index endoscopy; ongoing or recurrent hypotension and tachycardia after achieving haemodynamic stability; melaena or haematochezia after stool colour normalisation; and/or a fall in haemoglobin following the first endoscopy.
- All-cause mortality: Death occurring during the hospital admission following the index AUGIB event.
- Bleed-related mortality: Death occurring during the hospital admission, attributed directly to AUGIB.

### Data collection

Data were prospectively collected using standardised electronic case report forms adapted from the 2007 audit to reflect current practices (online supplemental appendix 1). Information on demographics, clinical presentations, laboratory values, management strategies (endoscopy, pharmacological interventions, transfusion practices) and outcomes (rebleeding, hospital stay, in-hospital mortality) was recorded. Data were collected until hospital discharge, death or 28 days of hospitalisation, with re-admissions tracked until 28 days post-discharge. Re-admissions during the study period were treated as new episodes. Further details are summarised in online supplemental methods.

### Statistical methods

Data are presented as frequencies with percentages or medians with IQRs. For comparisons between the 2022 and 2007 audits, differences in proportions were assessed using two-proportion Z-tests, based on published aggregate data from the 2007 audit.^4^ Differences in continuous variables, such as age, were evaluated using the Wilcoxon rank-sum test. Comparative analyses focused on differences in clinical management practices, such as endoscopy usage, transfusion practices and outcomes, including mortality and rebleeding rates, between the two audits.

Risk stratification scores (Glasgow-Blatchford and Rockall) were calculated for the 2022 cohort using raw data, where available, and reported with distributions, corresponding mortality, rebleeding rates, endoscopy usage and length of stay. Patient characteristics, endoscopy findings and mortality rates were stratified by mode of presentation and age group. Continuous variables were categorised where relevant to facilitate analysis and interpretation. Multivariate logistic regression was performed using the 2022 audit data to identify predictors of in-hospital mortality, adjusting for pre-endoscopy variables such as age, comorbidities, haemodynamic status, haemoglobin (Hb) level and clinical presentation features. Robust SEs were obtained after adjusting for clustering within hospital sites. Detailed patient characteristics are reported, but we categorised comorbidities into broader clinically relevant groups based on the organ systems involved to report crude mortality rates and for adjusted multivariate analysis. For example, ‘Cardiac disease’ encompasses patients with ischaemic heart disease or cardiac failure, while ‘Neurological disease’ includes those with stroke or dementia. These system-based groupings allow for more concise and interpretable analysis by reducing complexity and highlighting key clinical overlaps. This approach enabled clearer identification of patterns in comorbidity-related mortality outcomes.

Statistical analyses were conducted using R (V.4.1.3, R Foundation for Statistical Computing, Vienna, Austria) with a significance level set at p<0.05.

### Patient and public involvement

Patients and public were not involved in the design or conduct of this audit but inputs have been sought from a patient representative to design future work to improve AUGIB management.

## Results

A total of 147 NHS hospital sites across the UK contributed data, representing 127 of 147 eligible hospital trusts (response rate: 86%) providing data on 5141 AUGIB cases. Of these, 77.1% (3961) were new admissions with AUGIB, 20.3% (1044) developed AUGIB during a hospital stay and 2.1% (108) were transfers from other hospitals. Transfer cases included 49% from district general hospitals and 22% from non-acute hospitals. Transfer data were incomplete for 11% of cases.

### Patient characteristics

The cohort comprised 58.6% males, with a similar gender distribution across subgroups. The median age was 69 years (IQR: 54–80), with inpatients being older (median 74 years, IQR: 62–83). Comorbidities were present in 66.7% overall, more frequently in inpatients (79.9%) than new admissions (63.2%), with liver disease (18.9%) and ischaemic heart disease (18.0%) being most common. The median pre-endoscopy Rockall score was 2 (IQR: 1–4), and the Glasgow-Blatchford score (GBS) was 9 (IQR: 5–12), with inpatients showing higher scores (Rockall: 3 vs 2; GBS: 11 vs 8). These risk scores were calculated using the raw data captured as part of the audit. Detailed characteristics are summarised in table 1.

**Table 1:** Patient characteristics

Characteristics	All patients* n=5141 % (n)	New admissions n=3961 % (n)	Inpatients n=1044 % (n)	Transfers n=108 % (n)
Gender				
Male	58.6 (3014)	58.5 (2316)	60.2 (629)	54.6 (59)
Female	40.5 (2081)	40.9 (1619)	38.6 (403)	45.4 (49)
Missing	0.9 (46)	0.7 (26)	1.1 (12)	0
Age (years)				
Median (IQR)	69 (54–80)	68 (52–79)	74 (62–83)	68 (57–77.5)
Mean (SD)	66.1 (17.9)	64.7 (18.4)	71.4 (15.3)	66.2 (14.9)
Missing	0.9 (44)	0.7 (27)	0.9 (9)	0
Age group distribution years				
<60	32.9 (1693)	36.4 (1440)	20.1 (210)	35.2 (38)
60–79	39.4 (2025)	38.3 (1517)	43.3 (452)	42.6 (46)
≥80	26.8 (1379)	24.7 (977)	35.7 (373)	22.2 (24)
Comorbidities				
Any comorbidity	66.7 (3427)	63.2 (2505)	79.9 (834)	70.4 (76)
Ischaemic heart disease	18 (926)	16.7 (660)	23.4 (244)	19.4 (21)
Cardiac failure	10.7 (549)	9.3 (368)	16 (167)	11.1 (12)
Respiratory disease	14.8 (759)	13.4 (532)	20 (209)	15.7 (17)
Stroke	7.8 (401)	7 (279)	10.7 (189)	9.3 (10)
Dementia	4.7 (240)	4.5 (177)	5.8 (61)	1.9 (2)
Underlying haematological condition	3.7 (189)	3.5 (140)	4.5 (47)	1.9 (2)
Cancer/malignancy	13.9 (714)	13.1 (517)	18.2 (190)	6.5 (7)
Evidence of metastases	3.7 (190)	3.6 (142)	4.4 (46)	1.9 (2)
Renal disease	12.8 (656)	10.7 (423)	20.6 (215)	13.9 (15)
On renal replacement therapy	1.1 (55)	0.6 (24)	2.9 (30)	0.9 (1)
Documented liver disease	18.9 (972)	18.6 (737)	19.4 (203)	26.9 (29)
Alcohol-related cirrhosis	11.6 (596)	11.5 (456)	11.8 (123)	14.8 (16)
Non-alcohol aetiology cirrhosis	3.6 (184)	3.3 (129)	4.8 (50)	3.7 (4)
Chronic liver disease	2.2 (115)	2.4 (95)	1.7 (18)	0.9 (1)
Acute alcoholic hepatitis	0.5 (28)	0.6 (22)	0.5 (5)	0.9 (1)
Acute liver injury	0.1 (7)	0.1 (4)	0.3 (3)	0
Other	3.5 (179)	3.6 (143)	2.7 (28)	7.4 (8)
Haemodynamic status†				
Normal	60.2 (3095)	59.7 (1972)	61.1 (530)	60.2 (65)
Isolated tachycardia	25.9 (1330)	28.2 (1118)	17.5 (183)	25.9 (28)
Shock	5.5 (284)	5.5 (181)	6 (52)	4.6 (5)
Missing	8.4 (432)	5.5 (182)	15.8 (137)	9.3 (10)
Symptoms at presentation				
Fresh blood/haematemesis	30.6 (91575)	33.3 (1318)	21.2 (221)	30.6 (33)
Haematochezia/large volume bleeding PR	4.8 (247)	4.5 (184)	5.5 (47)	4.6 (5)
Melaena	57.1 (2935)	57.4 (2273)	55.9 (584)	70.4 (76)
Coffee ground vomit	20.3 (1045)	20.5 (812)	20.8 (217)	12 (13)
Shock/syncope	7 (359)	7.4 (295)	5.7 (60)	3.7 (4)
Other	11.8 (605)	11.3 (447)	13.6 (142)	14.8 (16)
Haemoglobin at presentation g/L				
>100	42.2 (2167)	46.2 (1829)	29.2 (305)	25 (27)
81–100	21.8 (1123)	20.4 (808)	27.5 (287)	23.1 (25)
71–80	11.5 (591)	10.4 (412)	15 (157)	20.4 (22)
≤70	19.9 (1022)	19 (754)	23 (240)	22.2 (24)
Median Hb (IQR)	95 (74–120)	99 (76–123)	86 (71–107)	83.5 (71–103)
Missing	4.6 (238)	4 (158)	5.3 (55)	9.3 (10)
Medications at presentation				
NSAIDs	7.4 (382)	8.6 (341)	3.4 (36)	4.6 (5)
Antiplatelets or anticoagulants	45.7 (2354)	39.8 (1577)	69.2 (722)	47.2 (51)
Antiplatelets	21.7 (1117)	19.7 (779)	30.3 (316)	19.4 (21)
Aspirin	15.4 (790)	13.8 (548)	21.9 (229)	11.1 (12)
P2Y12 (clopidogrel/prasugrel/ticagrelor) inhibitors	9.8 (505)	8.6 (341)	14.7 (153)	10.2 (11)
Both	3.5 (178)	2.8 (110)	6.3 (66)	1.9 (2)
Anticoagulants	30.6 (1573)	23.5 (929)	57.9 (604)	33.3 (36)
Warfarin	3.1 (160)	3.1 (123)	3.3 (34)	1.9 (2)
DOACs (apixaban/rivaroxaban/edoxaban/dabigatran)	18.2 (935)	17.8 (704)	19.4 (203)	23.1 (25)
LMWH or unfractionated heparin	10.1 (517)	2.9 (115)	37.6 (393)	8.3 (9)
Both antiplatelets and anticoagulants	6.5 (336)	3.3 (131)	19 (198)	5.6 (6)
Other				
History of alcohol use	30 (1540)	32.7 (1295)	20.1 (210)	27.8 (30)
Alcohol excess (>14 units/week)	19.4 (999)	21.5 (850)	12.2 (127)	15.7 (17)
Pre-endoscopy Rockall Score				
0–1	31.5 (1621)	34.9 (1382)	19.7% (206)	28.7% (31)
2–3	32.3 (1658)	32.1 (1271)	32.8% (342)	38.9% (42)
4–5	22.8 (1173)	22.4% (889)	24.6% (257)	23.1% (25)
6–7	4.4 (224)	4 (158)	6.3 (66)	0
Median (IQR)	2 (1–4)	2 (1–4)	3 (2–4)	2 (1–4)
Missing	9 (465)	6.6 (261)	16.6 (173)	9.3 (10)
Glasgow-Blatchford Score				
Low risk (0–1)	6.7 (342)	8.1 (320)	1.8 (19)	2.8 (3)
Medium risk (2–6)	21.4 (1098)	24.1 (956)	12.5 (131)	8.3 (9)
High risk (7–11)	33.4 (1715)	34 (1348)	30.6 (319)	42.6 (46)
Very high risk (≥12)	24.2 (1245)	22.1 (877)	32.1 (335)	27.8 (30)
Median (IQR)	9 (5–12)	8 (5–12)	11 (8–13)	10 (8–13)
Missing	14.4 (741)	11.6 (460)	23 (240)	18.5 (20)

* Includes 28 patients where mode of presentation was not recorded.

† Shock status was assessed on admission or based on the first set of observations after developing AUGIB. Shock is defined as a SBP <100 mm Hg, and heart rate ≥100. The ‘Missing’ category includes cases where either SBP or heart rate values were unavailable: SBP was missing in 412 patients, heart rate in 415 patients, either value in 432 cases and both values in 395 patients.

AUGIB, acute upper gastrointestinal bleeding; DOACs, direct oral anticoagulants; Hb, haemoglobin; LMWH, Low-molecular weight heparin; NSAIDs, non-steroidal anti-inflammatory drugs; PR, per rectum; P2Y12, purinergic receptor P2Y, G-protein coupled 12; SBP, systolic blood pressure.

### Presenting symptoms and haemodynamic status

Melaena, reported in 57.1% and haematemesis in 30.6% were the most common presenting symptoms. Shock, based on observations at time of presentation, was present in 5.5%, similarly distributed between new admissions and inpatients (table 1). A pre-endoscopic score was calculated and documented at presentation for 57.8% of the patients.

### Medications

At presentation, 30.6% of patients were on anticoagulants and 21.7% on antiplatelets, with direct oral anticoagulants (DOACs) being the most common (18.2%). Non-steroidal anti-inflammatory drug (NSAID) use was higher among new admissions (8.6%) than inpatients (3.4%) (table 1). Discontinuation of high-risk medications was common: NSAIDs were stopped in 91.3% (349/382), aspirin in 74.4% (588/790), purinergic receptor P2Y, G-protein coupled 12 (P2Y12) inhibitors in 84.9% (429/505), warfarin in 90% (144/160) and DOACs in 93.7% (876/935). Among patients on heparin, prophylactic doses were discontinued in 76.9% (310/403) and therapeutic doses in 80% (76/95).

### Pre-endoscopic management

A total of 10.5% patients were referred to higher level care on presentation, more frequently for inpatients (15.8%, 165/1044) than new admissions (8.9%, 351/3961). Overall, 5.8% were admitted to higher level care: 1.9% requiring Level II/high dependency unit (HDU) care and 3.9% requiring Level III/intensive therapy unit (ITU) care. Admission rates were higher among inpatients (2.4% to HDU and 6.3% to ITU) than for new admissions (1.7% and 3%, respectively) (table 2). Further details on reasons for non-admission and pre-endoscopic care are summarised in online supplemental material.

**Table 2:** Interventions and outcomes for AUGIB

Interventions and outcomes for AUGIB	Patients (n=5141) % (n)	New admissions (n=3961) % (n)	Inpatients (n=1044) % (n)
Admission under critical care	5.8 (297)	4.7 (186)	8.8 (92)
Level II/HDU	1.9 (97)	1.7 (66)	2.4 (25)
Level III/ITU	3.9 (199)	3 (120)	6.3 (66)
Resuscitation in the first 24 hours/pre-endoscopy			
Intravenous fluid (crystalloid)	68 (3497)	71.3 (2823)	57.4 (599)
Intravenous fluid (colloid)	0.8 (41)	0.6 (26)	1.3 (14)
Transfusion ≥1 unit			
Red blood cell transfusion (all)	49.8 (2561)	47.3 (1873)	59.2 (618)
Early red blood cell transfusion*	37.7 (1938)	35.8 (1420)	44.6 (466)
Median units (range)	2 (2–3)	2 (1–3)	2 (2–4)
FFP	5.4 (280)	4.9 (195)	7.1 (74)
Platelets	4.1 (208)	3.7 (145)	5.6 (58)
Major haemorrhage protocol	4.9 (251)	4.6 (182)	5.7 (59)
Endoscopy			
Inpatient endoscopy	83.2 (4279)	83.4 (3303)	83.1 (868)
1 endoscopy only	69.8 (3589)	71 (2814)	66.7 (696)
≥2 endoscopies	8.5 (437)	7.5 (296)	11.3 (118)
Missing information on no. of endoscopies	4.9 (253)	4.9 (193)	5.2 (54)
No inpatient endoscopy	16.8 (862)	16.6 (658)	16.9 (176)
Use of endoscopic therapy at index endoscopy	22.5 (1159)	21.7 (859)	25.5 (66)
Further bleeding (rebleeding) after index endoscopy	8.1 (414)	6.5 (258)	13 (136)
Surgery	0.7 (38)	0.8 (30)	0.6 (6)
IR	2.6 (133)	1.9 (74)	4.3 (45)
Median LOS (IQR)	5 days (3–9)	4 days (2–8)	10 days (5–17)
Re-admitted within 28 days	4 (205)	4.6 (181)	1.8 (19)
In-hospital all-cause mortality	8.8 (451)	5.7 (224)	20.3 (212)
Bleed-related mortality	3.7 (188)	2.6 (104)	7.3 (76)

* Early red blood cell transfusion defined as transfusion in the first 24 hours of presentation or pre-endoscopy whichever was shorter.

AUGIB, acute upper gastrointestinal bleeding; FFP, fresh frozen plasma; HDU, high dependency unit; IR, interventional radiology; ITU, intensive therapy unit; LOS, length of stay.

### Use of blood products

RBC transfusions were administered to 49.8% of patients, median of 2 units (range 2–3). Transfusion rates were higher among inpatients (59.2%, 618/1044) than new admissions (47.3%, 1873/3961). Early RBC transfusion (within 24 hours or pre-endoscopy) occurred in 37.7%, with similar trends between groups (table 2). Of these, 50.3% (975/1938) had a pretransfusion Hb level of ≤70 g/L, while 43% (833/1938) had Hb >70 g/L (table 3).

**Table 3:** Use of RBC transfusions for patients with AUGIB based on their pretransfusion Hb thresholds and haemodynamic status during the first 24 hours (early RBC) of their presentation

Pre transfusion Hb	Early RBC transfusion at this threshold N (n=1938)	Shock % (n)	Isolated tachycardia % (n)	Normal haemodynamic status % (n)	Missing data on haemodynamic status % (n)
Threshold of 70 g/L					
Hb≤70	975	9.1 (89)	22.8 (222)	61.5 (600)	6.6 (64)
Hb>70	833	10.3 (86)	26 (217)	56.5 (471)	7.1 (59)
Threshold of 80 g/L					
Hb≤80	1428	9.3 (133)	22.6 (323)	61.5 (878)	6.6 (94)
Hb>80	380	11 (42)	30.5 (116)	50.8 (193)	7.6 (29)
No Hb value	130	6.2 (8)	23.8 (31)	55.4 (72)	14.6 (19)

AUGIB, acute upper gastrointestinal bleeding; Hb, haemoglobin; RBC, red blood cell.

When applying a transfusion threshold of 70 g/L, 56.5% (471/975) of patients with Hb>70 g/L had normal haemodynamic status and were transfused. Similarly, at a threshold of 80 g/L, 50.8% (193/380) of patients with Hb>80 g/L and normal haemodynamic status received early RBC transfusions (table 3). Using a transfusion threshold of 70 g/L, at least 24% (471/1938) of the early RBC transfusions may have been inappropriate (table 4).

**Table 4:** Rebleeding and mortality rates based on early RBC transfusion appropriateness based on haemodynamic status and using thresholds of 70 g/L and 80 g/L

Pretransfusion Hb	Total numbers	Rebleeding % (n)	P value*	Mortality % (n)	P value*
Threshold of 70 g/L			0.49		0.74
Inappropriate transfusion	471	12.1 (57)		12.1 (57)	
Appropriate transfusion	1214	13.5 (164)		12.9 (156)	
Threshold of 80 g/L			0.62		0.24
Inappropriate transfusion	193	14.5 (28)		15.5 (30)	
Appropriate transfusion	1492	12.9 (193)		12.3 (183)	

Excludes patients with missing Hb values and missing haemodynamic status parameters (see table 3).

* χ2 test for p values between the group.

Hb, haemoglobin; RBC, red blood cell.

### Inpatient endoscopy and findings in AUGIB

Inpatient endoscopy was performed in 83.2% patients, most undergoing a single procedure (69.8%), and 8.5% required two or more. Endoscopic therapy at the index endoscopy was used in 22.5%, more commonly among inpatients (25.5%, 266/1044) than new admissions (21.7%, 859/3961) (table 2). Detection of endoscopic abnormalities declined with increasing age in both groups. Peptic ulcer disease (PUD) was the most common diagnosis, especially in older inpatients (27.5% in <60 years vs 45.2% in ≥80 years). Varices were more frequent in younger patients (16.5% of new admissions and 16.9% of inpatients <60 years), while malignancies were more common in older patients, reaching 5.7% among new-admissions ≥80 years (online supplemental table S1).

### Rebleeding

Rebleeding after the index endoscopy was reported in 8.1% of patients, with higher rates observed among inpatients (13%, 136/1044) compared with new-admissions (6.5%, 258/3961) (table 2). The overall median time to rebleeding was 2 days (IQR: 1–7). Rebleeding rates increased with pre-endoscopy risk scores (online supplemental table S2). Among patients who experienced rebleeding, in-hospital all-cause mortality was 26.6% (110/414), and bleed-related mortality was 17.6% (73/414).

Rebleeding rates were 12.1% for patients receiving inappropriate transfusions (defined as those given to patients with normal haemodynamic status above the respective threshold) and 13.5% for those receiving appropriate transfusions at a threshold of 70 g/L (p=0.49). At a threshold of 80 g/L, rebleeding rates were 14.5% and 12.9% for inappropriate and appropriate transfusions, respectively (p=0.62) (table 4). After adjustment for severity of bleed with the GBS, there were no significant differences in rebleeding for inappropriate transfusions at either threshold (70 g/L: adjusted OR (aOR) 0.92, 95% CI 0.64 to 1.33, p=0.65; 80 g/L: aOR 1.14, 95% CI 0.70 to 1.85, p=0.59) (online supplemental table S3).

### Surgery

Surgery was performed in 0.7% of cases, with a median patient age of 66 years (IQR: 52–74). Among these patients, 60.5% underwent one endoscopy prior to surgery, 18.4% had two or more endoscopies and 13.2% had no endoscopy before the intervention. 21% had undergone an IR procedure as well. The median time from presentation to surgery was 65.7 hours (IQR: 21–157.8 hours). The rate of surgical intervention was similar between new admissions (0.8%, 30/3961) and inpatients (0.6%, 6/1044) (table 2). Further details on indications and types of surgical procedures are summarised in online supplemental results. Postoperative mortality was 21% (8 deaths among 38 surgical procedures).

### Interventional radiology

IR was performed in 2.6% (133/5141) patients, with a median patient age of 71 years (IQR: 54–80). Among these patients, 56.4% underwent one prior endoscopy, 31.6% had two or more endoscopies and 9.8% had no endoscopy prior to the IR intervention. The median time from presentation to the IR procedure was 43.4 hours (IQR: 14–111.9 hours). IR was more frequent among inpatients (4.3%, 45/1044) compared with new-admissions (1.9%, 74/3961) (table 1). Further procedural details, bleeding control outcomes and modality types are summarised in the online supplemental results. Post-IR mortality was 19.5% (26 deaths among 133 procedures).

### Hospital stay

The median length of stay (LOS) was 5 days (IQR: 3–9), with shorter stays for new admissions (4 days, IQR: 2–8) compared with inpatients (10 days, IQR: 5–17). Within 28 days, 4% (205/5141) patients were readmitted, with higher readmission rates among new admissions (4.6%, 181/3961) compared with inpatients (1.8%, 19/1044) (table 2); LOS increased with higher pre-endoscopy risk scores.

### Mortality and related factors

The in-hospital all-cause mortality was 8.8%, higher among inpatients (20.3%, 212/1044) compared with new-admissions (5.7%, 224/3961). Bleed-related mortality occurred in 3.7% patients, with higher risks for inpatients (7.3%, 76/1044) than new admissions (2.6%, 104/3961) (table 2). Crude mortality varied by pre-endoscopic patient group and endoscopic diagnosis (table 5 and online supplemental table S4).

**Table 5:** Crude mortality rates and adjusted ORs for pre-endoscopy factors associated with mortality in patients with upper gastrointestinal bleeding

Pre-endoscopy factors	Crude mortality % (n)	Adjusted OR (95% CI)	P value
Age group (years)			
<60	5.1 (87/1693)	Reference	
60–79	9.3 (188/2025)	**1.78 (1.32 to 2.41)**	**<0.001**
≥80	12.4 (171/1379)	**2.32 (1.64 to 3.30)**	**<0.001**
Gender			
Male	9.1 (273/3014)	1.19 (0.97 to 1.46)	0.1
Female	8.3 (1722/2081)	Reference	
Symptoms at presentation			
Fresh blood/haematemesis	9.3 (147/1575)	1.30 (1 to 1.72)	0.05
Melaena	8.3 (243/2935)	0.87 (0.67 to 1.12)	0.27
Coffee ground vomit	7.9 (83/1045)	0.95 (0.68 to 1.31)	0.75
Haematochezia/large volume PR	10.5 (26/247)	1.09 (0.68 to 1.77)	0.70
Shock/syncope	13.4 (48/359)	**1.54 (1.08 to 2.24)**	**0.01**
Other	8.9 (54/605)	0.98 (0.68 to 1.38)	0.91
Haemodynamic status			
Normal	8.9 (277/3095)	Reference	
Tachycardia only	6.4 (85/1330)	0.78 (0.58 to 1.04)	0.1
Shock	18.3 (52/284)	**2.22 (1.53 to 3.17**)	**<0.001**
Comorbidities			
None	3.4 (58/1714)	Reference	
Cardiac disease	13.7 (176/1248)	**2.84 (1.95 to 4.21)**	**<0.001**
Renal disease	15.2 (100/656)	**3.80 (2.42 to 5.97)**	**<0.001**
Liver disease	13.7 (133/972)	**4.11 (2.81 to 6.08)**	**<0.001**
Respiratory disease	10.9 (83/759)	**1.97 (1.14 to 3.32)**	**0.01**
Neurological disease	10.5 (63/598)	**1.92 (1.03 to 3.44)**	**0.03**
Malignancy	15.3 (109/714)	**3.34 (2.09 to 5.33)**	**<0.001**
Other	17.8 (76/426)	1.36 (0.44 to 4.26)	0.6
Medications at presentation			
NSAIDs	3.1 (12/382)	**0.49 (0.25 to 0.96)**	**0.04**
Antiplatelets (aspirin/P2Y12)	8 (89/1117)	**0.68 (0.54 to 0.87)**	**0.002**
Anticoagulants (warfarin/DOACs/heparin)	13 (205/1573)	**1.43 (1.11 to 1.85)**	**0.006**
Haemoglobin at presentation (g/L)			
>100	6.1 (133/2167)	1	
71–100	10.7 (183/1714)	**1.38 (1.09 to 1.75)**	**0.008**
≤70	11.9 (122/1022)	**1.56 (1.15 to 2.11)**	**0.004**

Reference categories: Age group: <60 years; Gender: Female; Haemodynamic status: Normal; Haemoglobin: >100 g/L; Comorbidities: None; Medications: No NSAIDs, no antiplatelets, no anticoagulants; Symptoms: Absence of the specific symptom (eg, absence of fresh blood/haematemesis).

Crude mortality rates represent the proportion of deaths in each category without adjustment for other factors. Adjusted ORs were calculated using a multivariable logistic regression model accounting for age, gender, haemodynamic status, haemoglobin levels, symptoms, comorbidities and medications at presentation.

Haemodynamic status categories: Normal: Absence of shock or isolated tachycardia; Tachycardia only: SBP≥100 mm Hg and heart rate≥100 beats per minute; Shock: SBP<100 mm Hg and heart rate≥100 beats per minute.

ORs greater than 1 indicate increased odds of mortality compared with the reference category, while ORs less than 1 indicate reduced odds.

Statistically significant results (p<0.05) are bolded for emphasis.

DOACs, direct oral anticoagulants; NSAIDs, non-steroidal anti-inflammatory drugs; PR, per rectum; P2Y12, purinergic receptor P2Y, G-protein coupled 12; SBP, systolic blood pressure.

In a multivariable logistic regression model, aORs were calculated relative to a composite reference group comprising female patients aged <60 years with normal haemodynamic status, Hb >100 g/L, no comorbidities, no medications and absence of specific symptoms at presentation (table 5). Mortality increased with increasing age, from 5.1% in patients aged <60 years to 12.4% in those ≥80 years (aOR 2.32, p<0.001). Shock at presentation was associated with the highest crude mortality (18.3%) and more than double the odds of death (aOR 2.22, p<0.001). Comorbidities with the highest mortality included malignancy (15.3%), renal disease (15.2%) and liver disease (13.7%), all strongly associated with death (aORs: 2.84–4.11; all p<0.001). NSAID and antiplatelet use was associated with lower mortality (aOR 0.49 and 0.68), while anticoagulant use was associated with higher mortality (13%; aOR 1.43).

*Mortality based on endoscopic diagnoses:* Among patients with any abnormality identified during endoscopy, overall mortality was 8.5%; higher among inpatients (18.1%) compared with new admissions (5.8%). PUD had an overall mortality of 7.3%, while varices had a higher overall mortality (13.7%) (online supplemental table S4). Crude mortality also increased with higher pre-endoscopy risk scores (online supplemental table S2).

### Haemoglobin levels and transfusions

Crude mortality increased with decreasing Hb: 6.1% for >100 g/L, 10.7% for 71–100 g/L and 11.9% for ≤70 g/L, with aORs of 1.38 (1.09–1.75, p=0.008) and 1.56 (1.15–2.11, p=0.004), respectively. Furthermore, in patients receiving inappropriate transfusions (defined as those given to patients with normal haemodynamic status above the respective threshold), mortality was estimated as 12.1%, compared with 12.9% for those receiving appropriate transfusions at a threshold of 70 g/L (p=0.74). At a threshold of 80 g/L, mortality was estimated at 15.5% and 12.3% for inappropriate and appropriate transfusions, respectively (p=0.24) (table 4). After adjustment for GBS, there was no difference at 70 g/L (aOR 1.14, 95% CI 0.78 to 1.67, p=0.49), but at 80 g/L inappropriate transfusion was associated with higher mortality (aOR 1.60, 95% CI 1.00 to 2.56, p=0.05) (online supplemental table S3).

### Comparison of the 2007 and 2022 audits

Detailed results are shown in table 6. At a similar median age (68 years in 2007 and 69 years in 2022), the proportion of patients with at least one comorbidity increased from 50.2% in 2007 to 66.7% in 2022, with the prevalence of cirrhosis rising from 8.9% to 14.8%. NSAID and antiplatelet use decreased, whereas the use of anticoagulants more than doubled. The use of packed RBC transfusions increased from 43.3% in 2007 to 49.8% in 2022 (p<0.001).

**Table 6:** Comparative analysis on main outcomes with 2007 AUGIB audit

Period of data capture	2007 audit (n=6750) % (n)	2022 audit (n=5141) % (n)	Absolute difference (%)	95% CI (2022 to 2007)	P value
	2 months 2007	2 months 2022			
Median age	68 years (IQR 49–81)	69 years (IQR 54–80)	–	–	<0.001
Any (≥1) comorbidity	50.2 (3389)	66.7 (3427)	16.5	14.7 to 18.2	<0.001
Cirrhosis	8.9 (599)	14.8 (760)	5.9	4.7 to 7.1	<0.001
Medications at presentation					
NSAIDs	11.1 (751)	7.4 (382)	−3.7	−4.7 to −2.7	<0.001
Antiplatelets	33.1 (2233)	21.7 (1117)	−11.4	−12.9 to −9.8	<0.001
Anticoagulants	13.2 (889)	30.6 (1572)	17.4	15.9 to 18.9	<0.001
Inpatient endoscopy	74.1 (5004)	83.2 (4279)	9.1	7.6 to 10.6	<0.001
PUD	36.5 (1826/5004)	31.7 (1356/4279)	−4.8	−6.7 to −2.9	<0.001
Varices	10.9 (544/5004)	10.2 (437/4279)	−0.7	−1.9 to 0.6	0.30
Use of endoscopic therapy	23.4 (1172/5004)	27.1 (1159/4279)	3.7	1.9 to 5.4	<0.001
Further bleeding after index endoscopy	13.3 (668/5004)	9.7 (414/4279)	−3.7	−5 to −2.4	<0.001
Surgery	1.9 (127)	0.7 (38)	−1.1	−1.5 to −0.7	<0.001
IR	1.2 (84)	2.6 (133)	1.3	0.8 to 1.9	<0.001
Transfusion ≥1 unit					
Packed red blood cells	43.3 (2922)	49.8 (2561)	6.5	4.7 to 8.3	<0.001
Platelets	2.8 (189)	4.1 (208)	1.2	0.6 to 1.9	<0.001
FFP	7.4 (503)	5.4 (280)	−2	−2.9 to −1.1	<0.001
Median length of stay	6 days (IQR 2–16)	5 days (IQR 3–9)	–	–	<0.001
In-hospital mortality	10 (675)	8.8 (451)	−1.2	−2.3 to −0.2	0.02
New admission	6.8 (379/5547)	5.7 (224/3961)	−1.2	−2.2 to −0.2	0.02
Established inpatients	26.2 (288/1099)	20.3 (212/1044)	−5.9	−9.5 to −2.3	0.001

AUGIB, acute upper gastrointestinal bleeding; FFP, fresh frozen plasma; IR, interventional radiology; NSAIDs, non-steroidal anti-inflammatory drugs; PUD, peptic ulcer disease.

The proportion of patients undergoing inpatient endoscopy increased from 74.1% in 2007 to 83.2% in 2022. The use of endoscopic therapy increased from 23.4% to 27.1%, and the rate of further bleeding after index endoscopy decreased from 13.3% to 9.7% (p<0.001). Surgery rates decreased from 1.9% to 0.7% (p<0.001), while the use of IR increased from 1.2% to 2.6% (p<0.001).

## Discussion

This study provides a comprehensive update on the management and outcomes of AUGIB across the UK, 15 years after the 2007 national audit. The 2022 UK-wide AUGIB audit highlights substantial improvements in both patient outcomes and management practices compared with the previous UK audit and similar international studies.^4 20 21^ Between the two UK audits, in-hospital mortality reduced from 10.0% in 2007 to 8.8% in 2022, while rebleeding rates reduced from 13.3% to 9.7%. These advancements occurred despite an ageing patient population, an increase in comorbidities (50% to 67%) and shifts in medication use patterns, including reduced NSAID use (11% to 7.4%) and antiplatelet use (33% to 21.7%), alongside a doubling in anticoagulant use (13.0% to 30.6%). Additionally, the prevalence of cirrhosis increased from 9% to 15%, reflecting a growing number of patients at risk for variceal bleeding, consistent with broader global trends in liver disease prevalence.^22^

The improvements are likely driven by better access to endoscopic and IR services and evidence-based care pathways. In 2007, 74% patients underwent inpatient endoscopy, rising to 83% in the present study. Many international studies use endoscopy as an inclusion criterion, so it can be difficult to draw international comparisons.^20 21^ Where available, large international studies report inpatient endoscopy use in 89%—(Lu 2014)—suggesting improvement in the historic underuse and availability of endoscopy in the UK that is now more in keeping with practice in other countries.^23^ For example, access to 24/7 endoscopy has been available in Denmark since at least 2005.^20^ Initiatives following the 2007 UK AUGIB audit and results from the 2015 lower GIB audit have probably contributed to these improvements by raising awareness of pre-endoscopic management, enhancing service delivery and developing evidence-based care pathways for AUGIB management.^5^,^717^ These measures underscore the importance of systematic audits in driving progress in AUGIB management.

This study also underscores the increasing complexity of managing an ageing patient population with a higher burden of comorbidities. Observational studies in GIB consistently demonstrate that comorbidities are associated with poorer outcomes, particularly among established inpatients.^4 17 24^ Mortality among inpatients, while improved, remains high at 20.3% (down from 26.0% in 2007), compared with 5.6% for new admissions (down from 7.0% in 2007).^20^

The increasing use of anticoagulants further adds to complexity in managing patients with AUGIB. A systematic review of 3910 DOAC-treated patients with major GI bleeding estimated a 30-day mortality rate of 9% (95% CI 7% to 13%), highlighting the impact of anticoagulants on outcomes, although with heterogeneity due to variability in severity definitions.^25^ The evidence underscores that comorbidities and anticoagulant use increase the risk of rebleeding, mortality and length of stay, necessitating targeted interventions for these patients. Furthermore, the apparent protective association of NSAID use with mortality should be interpreted cautiously, as the absence of data on concurrent proton pump inhibitors (PPIs) use and the declining prevalence of *Helicobacter pylori* may have influenced the findings.^26 27^

A topic of special interest may be the use of transfusions. Following the 2007 audit showing that early RBC transfusion was associated with an increase in rebleeding risk and mortality, possibly due to the liberal transfusion strategies,^9^ subsequent randomised trials, including a UK cluster randomised trial (TRIGGER), demonstrated that restrictive transfusion strategies produced better, or at least non-inferior, outcomes compared with liberal strategies.^10^,^12^

In the 2022 audit, despite randomised controlled trial (RCT) evidence of possible harm from liberal RBC transfusion and guidelines recommending restrictive transfusion, RBC practices in AUGIB showed significant deviations.^10 12 28 29^ Nearly half of early transfusions (43.0%) were given at Hb≥70 g/L, with 24.3% of these classified as potentially inappropriate due to haemodynamic stability. These findings underscore variability in adherence to restrictive transfusion thresholds and UK guideline recommendations.^30^

In our exploratory analysis, inappropriate transfusion was not associated with higher rebleeding rates at either the 70 g/L or 80 g/L thresholds, even after adjustment for GBS. For mortality, no difference was observed at 70 g/L, whereas at 80 g/L inappropriate transfusion was associated with higher adjusted odds of death (aOR 1.60, 95% CI 1.00 to 2.56). These observations suggest a possible signal of harm at higher Hb thresholds; however, they must be interpreted cautiously given the observational design and the absence of sensitivity analyses excluding patients managed under a major haemorrhage protocol or accounting for comorbidities such as cardiac disease. More detailed analyses will be required to clarify these associations but are beyond the scope of the present manuscript.

However, this is not an isolated finding. A national audit in Canada found similarly variable compliance with transfusion-related recommendations.^23^ There are recognised limitations to the generalisability of the restrictive transfusion RCTs. The Villanueva ‘Barcelona’ trial excluded patients with massive exsanguination or acute coronary syndrome, while TRIGGER excluded only exsanguination.^10 12^ TRIGGER found no significant differences between treatment arms in further bleeding, all-cause mortality, interventions or length of stay, although as a pilot feasibility trial, it was not powered to assess outcomes.^10^ Notably, protocol violations were more common in the liberal arm (17% vs 4%), suggesting reluctance by physicians to commit patients to a liberal transfusion strategy. If such deviation and clinical uncertainty are seen in an RCT, they are even more likely to be seen in a multisite observational study. Reluctance to adopt restrictive transfusion may also stem from concern about cardiovascular disease, although a 2017 RCT in cardiac surgery demonstrated that a restrictive strategy was non-inferior to a liberal strategy for mortality.^31^ Taken together, RCT evidence favours a restrictive approach to transfusion, with liberal strategies consistently showing no benefit and, in some settings, possible harm. Restrictive transfusion is at least non-inferior, and in certain populations superior, to liberal transfusion with current international guidelines recommending a restrictive approach to transfusion in AUGIB.^29 32^ Restrictive transfusion also has important cost and resource implications, avoiding unnecessary use of a scarce blood product without improving outcomes.^33^

Future work should aim to define individualised transfusion thresholds based on patient demographics and comorbidities, supported by education and real-time decision support tools. A post-hoc TRIGGER analysis suggests optimal RBC thresholds may be higher than assumed.^10 34^ In patients with AUGIB with Hb>102.6 g/L, transfusion increased rebleeding and mortality versus no transfusion, underscoring the harm of unnecessary transfusion and the need for individualised plans over fixed cut-offs. The number of units transfused also consistently predicted adverse outcomes, highlighting the importance of personalised blood management.^35 36^ Protocol adherence to intravenous crystalloid resuscitation, observed in only 68% of cases, could further reduce transfusion demand.^37^ Outcomes also vary by AUGIB aetiology: restrictive strategies in ‘Barcelona’ trial improved survival in cirrhosis with Child-Pugh A/B (HR 0.30; 95% CI 0.11 to 0.85), but not Child-Pugh C (HR 1.04; 95% CI 0.45 to 2.37) and PUD bleeding (HR 0.70; 95% CI 0.26 to 1.25) compared with liberal transfusion.^12^ Further analyses from this multicentre study are planned.

Validated risk stratification tools, such as the GBS and pre-endoscopy Rockall Score, help identify low-risk patients suitable for outpatient care and stratify high-risk groups requiring closer monitoring.^38 39^ The GBS remains particularly valuable, with the BSG AUGIB care bundle and recent European Society of Gastrointestinal Endoscopy, American and international guidelines supporting outpatient management for patients with a GBS of 0–1.^29 32 37^ Some studies propose extending this low-risk threshold to GBS ≤2.^40 41^ However, in this audit, half of the low-risk patients (defined as GBS 0–1) underwent inpatient endoscopy with a median LOS of 2 days (IQR: 1–4). This group had rebleeding and mortality rates below 2%, suggesting many could have safely avoided admission in favour of outpatient care. It is likely that some truly low-risk patients were discharged directly from the emergency department or primary care and thus not captured. Those admitted with GBS 0–1 may have had other clinical indications, potentially inflating mortality rates. Conversely, high-risk patients (GBS≥12) had markedly higher rebleeding (15%) and mortality rates (17%), underscoring the importance of early risk assessment to guide interventions, reduce unnecessary admissions and optimise resources.

Endoscopy utilisation rose from 74% in 2007 to 83% in 2022, reflecting better access and impact of updated guidelines emphasising timely diagnostic and therapeutic procedures.^28 29 37 42^ PUD remains the most common finding, though its prevalence has decreased, likely due to increased use of PPIs and improved management of *H. pylori*.^26 27^ Conversely, variceal bleeding rates have remained stable but are associated with persistently high inpatient mortality (29.7% in 2022 vs 41% in 2007). Notably, about one-third of patients undergoing endoscopy had no abnormalities identified, and only 27% required therapeutic interventions, further stressing the need for improved patient selection.

Use of endoscopic therapy has increased (27.1% in 2022 vs 23.0% in 2007), reflecting advancements in haemostatic techniques and increased endoscopist expertise.^13^ It is difficult to draw a direct comparison with other studies as many do not directly report use of endotherapy, although it can be inferred from the reporting of high-risk stigmata at endoscopy. International rates of 40–51% are reported, suggesting that the UK rate remains low.^20 21^ This may be due to methodological differences in the studies or reflect an ongoing difference in the availability of endotherapy. Concurrently, rates of further bleeding after index endoscopy have decreased (9.7% vs 13.3%), indicating improvements in haemostatic interventions and post-endoscopy care. However, concerns remain around endoscopy training and appropriate selection of choice of endotherapy. A recent UK survey reported limited exposure to haemostatic techniques and low confidence among trainees, even at senior levels.^43^ Investment in simulation-based training, emergency service integration and multidisciplinary education is essential to sustain improvements in AUGIB outcomes.

Post-endoscopic management increasingly relies on IR and selective surgery for persistent or recurrent bleeding. Surgical interventions have decreased to 0.7%, while IR use stands at 2.6%, often as a secondary intervention where endoscopy is insufficient. This shift highlights IR’s role in achieving haemostasis for complex cases, underscoring the need for accessible IR services, especially in high-volume centres.

Although the study observed increased use of interventions such as endoscopy, blood transfusion and interventional radiology, the overall reduction in median LOS was limited to 1 day. However, hospital stay may be influenced by factors unrelated to AUGIB, including patient comorbidities, social care requirements and discharge planning delays, particularly relevant within the UK healthcare system.^44^ While cost data were not collected, future studies should evaluate the cost-effectiveness of current AUGIB management strategies, taking these broader system-level factors into account.

Strengths of this study include its prospective design and alignment with established audit frameworks from 2007 and 2015.^4 17^ Using similar methodologies, including time frames, while updating the questionnaire for current practice, the audit provides a robust benchmark for AUGIB management trends. Participation from 147 NHS sites, spanning academic centres and smaller hospitals across diverse regions, offers a comprehensive snapshot of UK practice. The detailed questionnaire captured pre-endoscopic, peri-endoscopic and post-endoscopic management. Although participation was lower in 2022 (147 vs 208 in 2007), reflecting trust mergers and integrated care systems,^39 40^ the audit remains nationally representative, though site-level comparisons are not feasible.

Several limitations must be acknowledged. Challenges with information technology (IT) systems, clinician workload and COVID-19 delays may have affected data completeness. Some NHS hospitals, including a few high-volume centres, did not participate and some variables had missing data despite strict validation. Variability in supervision and trainee handover may also have influenced accuracy. Reliance on goodwill and lengthy questionnaires may have contributed to under-reporting, though the large sample still provides a representative dataset. These operational challenges highlight the need for stronger IT systems, clearer guidelines and enhanced training to streamline future audits.

## Conclusions

This study highlights advances in AUGIB management, particularly in endoscopy and IR, while recognising challenges in addressing high-risk populations and optimising resources. Transfusion remains an area of inconsistent practice likely due to underlying clinical uncertainty of how to apply findings from previous randomised studies. Use of validated risk scores, improving education and training programmes, particularly with regard to endoscopic intervention, and expanding multidisciplinary care could drive further improvements. These findings support quality improvement initiatives for equitable high-quality AUGIB care across the UK and offer lessons applicable to improving AUGIB management internationally.

## Supplementary material

10.1136/gutjnl-2025-335134 online supplemental file 1

10.1136/gutjnl-2025-335134 online supplemental file 2

10.1136/gutjnl-2025-335134 online supplemental file 3

10.1136/gutjnl-2025-335134 online supplemental file 4

## Data Availability

Data are available upon reasonable request.
