# Supplementary material for: Acute upper gastrointestinal bleeding in the UK: 2022 audit update
Source: Gut. 2025 Nov 19;75(4):e335134. doi: 10.1136/gutjnl-2025-335134 (PMC13018800; doi:10.1136/gutjnl-2025-335134)
Supplement: online supplemental file 1 [file gutjnl-75-4-s001.docx]

### ****SUPPLEMENT TO**** Acute upper gastrointestinal bleeding in the UK: 2022 Audit Update

First Autor

Gaurav B Nigam; Translational Gastroenterology and Liver Unit, Oxford University Hospitals NHS Trust, Oxford, United Kingdom.

**Supplementary Methods:** A unique code ensured data anonymity, with cases entered into a secure online database accessible via site-specific passwords. Periodic validation ensured data completeness, and audit leads resolved inconsistencies or missing information. The central team monitored inactive cases weekly, prompting completion by local teams. Paper versions of forms were available for sites with limited digital access.

Data Handling: Data were anonymised and stored in a secure database. Validation checks included cross-referencing data entries with source documentation, verifying logical consistency between related variables, and addressing any discrepancies flagged during routine quality control processes. Cases were tracked until discharge, death, or 28 days of hospitalisation, with follow-up for readmissions occurring within the audit period. Missing data, where applicable, are reported for each variable.

Transfer data were analysed descriptively due to the small sample size and the lack of detailed information on patient presentation at the initial hospital, which precluded further analysis.

**Supplementary Results:**

*Pre-endoscopy discontinuation of high-risk medications*: Among patients taking NSAIDs (median age 63 years, IQR 50–75), clinicians discontinued the drug in 91.3% (349/382) of cases. For aspirin users (n=790, median age 75 years, IQR 66–82), 74.4% (588/790) had the medication stopped, and for those on P2Y12 inhibitors (n=505, median age 75 years, IQR 67–82), therapy was discontinued in 84.9% (429/505). Among patients on warfarin (n=160, median age 78 years, IQR 68–85), 90% (144/160) had the anticoagulant stopped. Similarly, for DOAC users (n=935, median age 80.5 years, IQR 74–86), these were discontinued for 93.7% (876/935) of cases. For patients on heparin (n=517, median age 73 years, IQR 60–83), discontinuation rates were 76.9% (310/403) for prophylactic doses, 80% (76/95) for therapeutic doses, 63.6% (7/11) for bridging therapy, and 45.4% (5/11) for cases with missing dose information.

*Reasons for no admission for higher level care:* Among the patients referred for higher level care but not admitted, the reasons for non-admission included clinical assessment deeming admission unnecessary (67%), lack of suitability for critical care due to comorbidities (26%), and other reasons (7%). These trends were consistent across new admissions and inpatients.

*Additional pre-endoscopic management:* In the first 24 hours prior to endoscopy, intravenous crystalloid fluids for initial resuscitation were administered to 68% (3497/5141) of patients. Crystalloids were more commonly used among new admissions (71.3%, 2823/3961) compared to inpatients (57.4%, 599/1044). The mean volume of crystalloids administered overall was 941 ml (SD 420 ml). Intravenous colloid fluids were used in a much smaller proportion of patients (0.8%, 41/5141), with slightly higher usage among inpatients (1.3%, 14/1044) compared to new admissions (0.6%, 26/3961). The overall mean volume of colloids administered was 641 ml (SD 435 ml).

*Surgical Indications and Procedures:*

The primary reason for surgery was uncontrolled bleeding (52.6%), followed by malignancy (26.3%), peritonitis or perforation (5.3%), and stigmata of recent haemorrhage or high-risk lesions (2.6%). Other reasons accounted for 21.1%, categorised as miscellaneous “free text.”

Common surgical procedures included partial or other gastrectomy (15.8%), duodenotomy with underrunning of the vessel (13.2%), and oversewing or plication of ulcers (13.2%). Less frequent interventions included pyloroduodenotomy with underrunning of the vessel (5.3%), laparoscopic or open wedge excision of the bleeding lesion (5.3%), and gastroduodenal artery ligation (2.6%). Postoperative mortality was 21% (8 deaths among 38 surgical procedures).

*Interventional Radiology Procedures:*

Diagnostic CT angiography alone was performed in 45.9% of cases, with 13.1% experiencing further bleeding after the procedure. Diagnostic and therapeutic angiography was performed in 39.1% patients, with a 90.4% success rate in controlling bleeding; however, further bleeding occurred in 15.4%. Empiric embolisation, based on findings from prior endoscopy or CT angiography, was used in 7.5% of patients, achieving a 90% success rate in controlling bleeding but with a further bleeding rate of 30%. TIPSS alone was performed in 4.5% patients, achieving haemostasis in 83.3%, with further bleeding reported in 16.7%.

**SUPPLEMENTARY TABLES:**

**Supplementary Table S1**: Main endoscopic diagnoses by age group and type of presentation

| Endoscopic diagnoses on 1^st^ or 2^nd^ endoscopy | New-admissions (n= 3961) | | | Inpatients (n=1044) | | | Total 4279* |
| --- | --- | --- | --- | --- | --- | --- | --- |
|  | Age <60 years  %(n) | Age 60-79 years  %(n) | Age ≥ 80 years  %(n) | Age <60 years  %(n) | Age 60-79 years  %(n) | Age ≥ 80 years  %(n) |  |
| Any abnormality | 71.9% (844) | 68% (900) | 56.7% (445) | 71.9% (128) | 70.6% (271) | 64.8% (195) | 67.1% (2872) |
| Peptic ulcer | 29.9% (351) | 30.4% (402) | 31% (243) | 27.5% (49) | 33.9% (130) | 45.2% (136) | 31.7% (1356) |
| Varices | 16.5% (194) | 10.6% (141) | 1.9% (15) | 16.9% (30) | 7.6% (29) | 1.7% (5) | 10.2% (437) |
| Malignancy | 2.4% (28) | 5.3% (70) | 5.7% (45) | 2.2% (4) | 2.1% (8) | 1.7% (5) | 3.8% (163) |
| Oesophagitis | 17.4% (204) | 15.9% (211) | 12% (94) | 19.7% (35) | 23.2% (89) | 18.9% (57) | 16.5% (704) |
| Mallory-Weiss tear | 3.7% (43) | 2.1% (28) | 1.3% (10) | 1.7% (3) | 1.3% (5) | 0.3% (1) | 2.2% (92) |
| Portal hypertensive gastropathy (PHG) | 7.8% (92) | 4.8% (64) | 0.8% (6) | 9% (16) | 4.4% (17) | 0.7% (2) | 4.7% (203) |
| Dieulafoy lesion | 0.8% (9) | 1.1% (14) | 1.5% (12) | 1.1% (2) | 1.6% (6) | 2.3% (7) | 1.2% (51) |
| Gastric antral vascular ectasia | 0.8% (9) | 3.1% (41) | 2.2% (17) | 1.1% (2) | 3.4% (13) | 0.7% (2) | 2% (87) |
| Telangiectasia | 1% (12) | 2% (26) | 2.2% (17) | 1.1% (2) | 2.6% (10) | 1% (3) | 1.7% (73) |
| Post-sphincterotomy bleed | 0.1% (1) | 0.1% (1) | 0.5% (4) | 0.6% (1) | 0.8% (3) | 0.3% (1) | 0.3% (12) |
| Other | 8.3% (97) | 9.9% (131) | 9.9% (78) | 13.5% (24) | 12.2% (47) | 9.3% (28) | 9.7% (414) |
| No abnormality noted | 28.1% (330) | 32% (424) | 43.3% (340) | 28.1% (50) | 29.4% (113) | 35.2% (106) | 32.9% (1407) |

**Footnotes:** Patients may have multiple diagnoses, and each diagnosis is captured only once per patient.

*Includes 108 transfers from other hospitals or admission data missing

**Supplementary Table S2: Glasgow-Blatchford and Pre-Endoscopy Rockall risk score categories (from raw data where available) and outcomes**

| Pre-endoscopy risk score | Cases  % (n) | Endoscopy performed  % (n) | Re-bleeding in those undergoing endoscopy  % (n) | Crude Mortality rate  % (n) | Median length of stay (IQR)  in days |
| --- | --- | --- | --- | --- | --- |
| **Glasgow-Blatchford Score** |  |  |  |  |  |
| Low risk (0–1) | 6.7% (342) | 55.8% (191) | 1.6% (3) | 1.5% (5) | 2 (1–4) |
| Medium risk (2–6) | 21.4% (1098) | 80.7% (886) | 4.5% (40) | 4.5% (49) | 4 (2–7) |
| High risk (7–11) | 33.4% (1715) | 89.2% (1529) | 9.6% (147) | 7% (120) | 5 (3–9) |
| Very high risk (≥12) | 24.2% (1245) | 89.5% (1114) | 15.4% (172) | 16.9% (211) | 7 (4–13) |
| Missing | 14.4% (741) | 75.4% (559) | 9.3% (52) | 8.9% (66) | 5.5 (3–11) |
| **Pre-endoscopy Rockall Score** |  |  |  |  |  |
| 0–1 | 31.5% (1621) | 82.4% (1336) | 7.4% (99) | 2.3% (38) | 4 (2–7) |
| 2–3 | 32.3% (1658) | 85.1% (1411) | 9.9% (139) | 9.4% (156) | 6 (3–11) |
| 4–5 | 22.8% (1173) | 85% (997) | 11.4% (114) | 13.7% (161) | 6 (3–10) |
| 6–7 | 4.4% (224) | 79.9% (179) | 14.5% (26) | 24.1% (54) | 8 (5–13) |
| Missing | 9% (465) | 76.6% (356) | 10.1% (36) | 9% (42) | 6 (2.2–13) |

**Supplementary Table S3: Adjusted odds of rebleeding and mortality according to appropriateness of early red blood cell transfusion at haemoglobin thresholds of 70 g/L and 80 g/L**

| Pre- transfusion Hb | Group | Rebleeding | | Mortality | |
| --- | --- | --- | --- | --- | --- |
|  |  | OR (95% CI) | p-value | OR (95% CI) | p-value |
| Threshold of 70 g/L |  |  |  |  |  |
|  | Appropriate transfusion | 1 (reference) | - | 1 (reference) | - |
|  | Inappropriate transfusion | 0.92 (0.64-1.33) | 0.65 | 1.14 (0.78-1.67) | 0.49 |
| Threshold of 80 g/L |  |  |  |  |  |
|  | Appropriate transfusion | 1 (reference) | - | 1 (reference) | - |
|  | Inappropriate transfusion | 1.14 (0.70-1.85) | 0.59 | 1.60 (1.00-2.56) | 0.05 |

Odds ratios adjusted for Glasgow-Blatchford Score (GBS) and clustered by site. Appropriate transfusion is the reference category.

**Supplementary Table S4 : Crude mortality rates by endoscopic diagnosis**

| Endoscopic diagnoses on 1^st^ or 2^nd^ endoscopy | Crude mortality rates | | |
| --- | --- | --- | --- |
|  | Total* | New-admissions | Inpatients |
| Any abnormality | 8.5%% (243/2872) | 5.8%% (128/2202) | 18.1% (108/598) |
| Peptic ulcer | 7.3% (99/1356) | 4.5% (45/1003) | 16.4% (52/317) |
| Varices | 13.7% (60/437) | 10.5% (37/352) | 29.7% (19/64) |
| Malignancy | 10.4% (17/163) | 9.7% (14/144) | 11.8% (2/17) |
| Oesophagitis | 7.8% (55/704) | 4.5% (23/512) | 17.6% (32/182) |
| Mallory-Weiss tear | 6.5% (6/92) | 6.1% (5/82) | 11.1% (1/9) |
| Portal hypertensive gastropathy (PHG) | 9.4% (19/203) | 9.3% (15/162) | 8.6% (3/35) |
| Dieulafoy lesion | 13.7% (7/51) | 5.7% (2/35) | 33.3% (5/15) |
| Gastric antral vascular ectasia | 6.9% (6/87) | 4.5% (3/67) | 16.7% (3/18) |
| Telangiectasia | 2.7% (2/73) | 3.6% (2/56) | 0% (0/15) |
| Post-sphincterotomy bleed | 0% (0/12) | 0% (0/6) | 0% (0/5) |
| Other | 9.7% (40/414) | 6.5% (20/309) | 19.2% (19/99) |
| No abnormality noted | 5.6% (79/1407) | 3.3% (36/1101) | 14.4% (39/270) |

Footnotes: Patients may have multiple diagnoses, and each diagnosis is captured only once per patient. The rates presented are crude mortality estimates and are not adjusted for overlap among diagnoses or potential confounders such as age, comorbidities, or severity of bleeding.

*Includes 108 transfers from other hospitals or admission data missing
