# Supplementary material for: Acute upper gastrointestinal bleeding in the UK: 2022 audit update
Source: Gut. 2025 Nov 19;75(4):e335134. doi: 10.1136/gutjnl-2025-335134 (PMC13018800; doi:10.1136/gutjnl-2025-335134)
Supplement: online supplemental file 2 [file gutjnl-75-4-s002.pdf]

National Comparative Audit of Blood Transfusion

BRITISH SOCIETY OF GASTROENTEROLOGY

NHS Blood and Transplant

UK Comparative Audit of Acute Upper Gastrointestinal Bleeding (AUGIB) and the use of Blood

Patient Audit Booklet

Patient Number

Site Code

BASL British Association for the Study of the Liver

Royal College of Physicians

This page is intentionally blank, please use this space to write any notes you may find helpful during the audit.

SECTION ONE - DEMOGRAPHICS

1. What is the patient's age at presentation? ☐☐☐

2. What is the patient's gender? ☐ Female ☐ Male

3. What is the patient's ethnicity?

White

☐ White English, Welsh, Scottish, Northern Irish or British

☐ White Irish

☐ White Gypsy or Irish Traveller

☐ White – other

Mixed or Multiple ethnic groups

☐ White and Black Caribbean

☐ White and Black African

☐ White and Asian

☐ Other mixed or multiple ethnic background

Asian or Asian British

☐ Indian

☐ Pakistani

☐ Bangladeshi

☐ Chinese

☐ Other Asian background

Black, African, Caribbean or Black British

☐ African

☐ Caribbean

☐ Other Black, African or Caribbean background

Other ethnic group

☐ Arab

☐ Other ethnic group

SECTION TWO – WHEN & WHERE THE PATIENT PRESENTED

4. Where did the patient present with AUGIB?

☐ Presented to hospital with AUGIB *Now answer Q4a*

☐ AUGIB in patient hospitalised for other reason *Now answer Q4b*

☐ Transfer from another hospital for the management of AUGIB *Now answer Q4c*

4a. Date & time of presentation to ED/A&E with AUGIB

☐☐ ☐☐ 2 0 2 2   ☐☐ ☐☐

4b. Date & time of presentation with the inpatient AUGIB

☐☐ ☐☐ 2 0 2 2   ☐☐ ☐☐

4c. Type of hospital from which the patient was transferred

☐ Non-acute hospital

☐ Small DGH

☐ Independent hospital

☐ Other

4c(i) Date & time of transfer to your hospital

☐☐ ☐☐ 2 0 2 2   ☐☐ ☐☐

4c (ii). When did the patient present at the hospital that referred the patient to you?

☐ Details about presentation at referring hospital are not available

Date & time of presentation with AUGIB

☐☐ ☐☐ 2 0 2 2   ☐☐ ☐☐

SECTION THREE – OBSERVATIONS ON PRESENTATION WITH AUGIB

5. What were the observations on presentation?

☐ Observations are not available

Temperature ☐

Heart rate ☐

Systolic BP ☐

Diastolic BP ☐

Respiratory rate ☐

Oxygen saturation ☐

Room air or supplemental O_2 *(select one that is applicable)*

☐ Room air ☐ Supplemental Oxygen

Level of consciousness (use ACVPU) ☐

SECTION FOUR – HOW THE PATIENT PRESENTED

6. How did the patient present with their AUGIB?

☐ Fresh blood / Haematemesis

☐ Haematochezia / Large volume bleeding PR

☐ Melaena

☐ Coffee ground vomit

☐ Shock / Syncope

☐ Other, please state

6a. Other details

☐

7. Was AUGIB care bundle completed within the first 24 hours of presentation?

☐ Yes ☐ No ☐ No clear documentation

SECTION FIVE – CO-MORBIDITIES & ONGOING MEDICATIONS at time of presentation with AUGIB

8. What was the ASA status of the patient?

☐ I

☐ II

☐ III

☐ IV

☐ Not documented

9. Did the patient have any of the following clinically significant comorbidities?

☐ Ischaemic heart disease

☐ Cardiac failure

☐ Respiratory disease

☐ Stroke

☐ Dementia

☐ Underlying haematological condition

☐ Cancer / malignancy

　　☐ *Evidence of metastases*

☐ Renal disease

　　☐ *Patient on renal replacement therapy*

☐ Documented liver disease *Now answer Q9a*
If you did not tick “Documented liver disease” go to Question 22

9a. Diagnosis for underlying liver disease: *Select all that apply*

☐ Alcohol-related cirrhosis

☐ Cirrhosis, non-alcohol aetiology

☐ Chronic liver disease

☐ Acute alcoholic hepatitis

☐ Acute liver injury

☐ Other, please state

9b. Other details

10. Do / did they have ascites? *(During hospital admission for current episode of AUGIB)*

☐ Yes *Now answer Q10a* ☐ No ☐ Not documented

10a. Severity of ascites

☐ Mild or suppressed with medication

☐ Moderate to severe, or refractory

11. Do / did they have encephalopathy? *(During hospital admission for current episode of AUGIB)*

☐ Yes *Now answer Q11a* ☐ No ☐ Not documented

11a. Grade as per West-Haven criteria

☐ Grade 1: Lack of awareness, euphoria or anxiety, shortened attention span, impaired performance of addition.

☐ Grade 2: lethargy or apathy, minimal disorientation for time or place, subtle personality change, inappropriate behaviour, impaired performance of subtraction.

☐ Grade 3: somnolence to semi stupor but responsive to verbal stimuli, confusion, gross disorientation.

☐ Grade 4: coma (unresponsive to verbal or noxious stimuli).

12. Is the patient under regular follow-up for the underlying liver condition?

☐ Yes *Now answer Q12a* ☐ No

12a. Patient is seen at follow-up by

☐ Gastroenterologist

☐ Hepatologist

☐ Other, please state

12b. Other details

13. Is the patient on a liver transplantation waiting list?

☐ Yes ☐ No

Before this episode of bleeding

14. Was the patient on a variceal band ligation programme?

☐ Yes *Now answer Q15*   ☐ No   ☐ Don't know

15. Has the patient undergone variceal banding?

☐ Yes *Now answer Q15a & b*   ☐ No

15a. Date & time of last band ligation

☐☐ ☐☐ ☐☐☐☐   ☐☐ ☐☐

15b. Date & time of last endoscopy

☐☐ ☐☐ ☐☐☐☐   ☐☐ ☐☐

16. Which best describes this presentation?

☐ Index presentation with AUGIB *Please go to Q21*

☐ Repeat presentation with AUGIB *Now answer Q17*

17. How many previous variceal bleeding episodes did the patient have?

☐

Thinking about the last variceal bleeding episode

18. Details of last variceal bleed

☐ Oesophageal bleed

☐ Gastric bleed

☐ Other, please state

18a. Other details

☐

19. Date of last variceal bleed ☐☐ ☐☐ ☐☐☐☐

20. Was a referral for TIPSS made? ☐ Yes *Now answer Q20a* ☐ No
(For the last variceal bleeding episode)

20a. Was TIPSS done? ☐ Yes *Now answer Q20b* ☐ No

20b. Date when TIPSS was done ☐☐ ☐☐ ☐☐☐☐

21. On current hospital admission, was the patient on prophylaxis with beta blockers?

☐ Yes *Now answer Q21a* ☐ No *Now answer Q22*

21a. Select drug and give details of dose

☐ Carvedilol	Dose (mg)	☐	☐ OD	☐ BD
☐ Propranolol	Dose (mg)	☐	☐ OD	☐ BD
☐ Nadolol	Dose (mg)	☐	☐ OD	☐ BD

22. For current episode of AUGIB, did the patient require a Sengstaken-Blakemore / Minnesota / Linton tube in place prior to the endoscopy?

☐ Yes ☐ No

23. Was the patient on aspirin? ☐ Yes *Now answer Q23a* ☐ No

23a. Was it discontinued? ☐ Yes *Now answer Q23b* ☐ No

23b. Date & time discontinued or last taken by patient

☐☐ ☐☐ 2 0 2 2 ☐☐ ☐☐

24. Was the patient on P2Y12 inhibitors? ☐ Yes *Now answer Q24a* ☐ No
(Clopidogrel, Prasugrel or Ticagrelor)

24a. Was it discontinued? ☐ Yes *Now answer Q24b* ☐ No

24b. Date & time discontinued or last taken by patient

☐☐ ☐☐ 2 0 2 2 ☐☐ ☐☐

25. Was the patient on warfarin? ☐ Yes *Now answer Q25a* ☐ No

25a. Was it discontinued? ☐ Yes *Now answer Q25b* ☐ No

25b. Date & time discontinued or last taken by patient

						2	0	2	2						

26. Was the patient on direct oral anticoagulants? ☐ Yes *Now answer Q26a* ☐ No
(Apixaban, Rivaroxaban, Edoxaban, Dabigatran)

26a. Was it discontinued? ☐ Yes *Now answer Q26b* ☐ No

26b. Date & time discontinued or last taken by patient

						2	0	2	2						

27. Was the patient on low molecular weight heparin or unfractionated heparin?

☐ Yes *Now answer Q27a & b* ☐ No

27a. Why were they given heparin?

☐ Prophylaxis

☐ Therapeutic dose

☐ Bridging therapy

27b. Was it discontinued? ☐ Yes *Now answer Q27c* ☐ No

27c. Date & time discontinued or last given to patient

						2	0	2	2						

28. Was the patient on NSAIDs? ☐ Yes *Now answer Q28a* ☐ No

28a. Was it discontinued? ☐ Yes *Now answer Q28b* ☐ No

28b. Date & time discontinued or last taken by patient

						2	0	2	2						

29. Is there a history of regular alcohol consumption?

☐ Yes *Now answer Q29a* ☐ No

29a. How many units per week? ☐

SECTION SIX - PRE-ENDOSCOPY RISK ASSESSMENT & INITIAL MANAGEMENT

30. Was a pre-endoscopy risk score calculated?

☐ Yes *Now answer Q30a* ☐ No

30a. Which score was used?

☐ Glasgow – Blatchford Score

☐ Pre-endoscopy Rockall Score

☐ Other

31. Was a referral for critical care review made?

☐ Yes *Now answer Q31a* ☐ No *Now answer Q32*

31a. Did the patient require admission under the critical care team?

☐ Yes *Now answer Q31b* ☐ No *Now answer Q31c*

31b. What was the level of admission? Select one option

☐ Level II / HDU

☐ Level III / ITU

31c. Why did the patient not require admission?

☐ Clinically not required as per assessment from critical care

☐ Not suitable for critical care admission in view of co-morbidities

☐ Other, please state

32. Was the Major Haemorrhage Protocol activated for this episode of AUGIB?

☐ Yes ☐ No ☐ Not documented

33. Which of the following did the patient receive for the AUGIB as part of initial resuscitation in the first 24 hours (pre-endoscopy)? *Select all that apply*

☐ Intravenous fluid (crystalloid) *Now answer Q34a*

☐ Intravenous fluid (colloid) *Now answer Q34b*

☐ Red blood cell transfusion *Now answer Q35*

☐ Fresh frozen plasma

☐ Platelets

☐ Human Albumin Solution

☐ Other

☐ None of the above

34a. What was the volume and rate at which the first IV crystalloid was given?

i. Volume in mL ☐

ii. Rate (mL/Hour) ☐

34b. What was the volume and rate at which the first IV colloid was given?

i. Volume in mL ☐

ii. Rate (mL/Hour) ☐

35. How many units of red blood cells were transfused within 24 hours of presentation (pre-endoscopy) for AUGIB?

☐

36. Were any of the following used within the first 24 hours (pre-endoscopy) after AUGIB?

☐ Vitamin K

☐ Cryoprecipitate

☐ Fibrinogen concentrate

☐ Prothrombin Concentrate Complex

☐ Recombinant factor VIIa

☐ Tranexamic acid

☐ Other

☐ None of the above

37. Which of the following drugs were started in hospital as a treatment for AUGIB before endoscopy or before diagnosis was made? *Select all that apply*

☐ PPI oral

☐ PPI intravenous boluses

☐ PPI intravenous infusion

☐ Terlipressin

☐ Octreotide

☐ Prokinetic agents

☐ IV antibiotics

☐ Other

☐ None of the above

38. Was an upper GI endoscopy performed for this episode of AUGIB?

☐ Yes *Now answer Q39* ☐ No *Now go to Q72*

39. Date & time of referral for endoscopy

☐☐ ☐☐ 2 0 2 2 ☐☐ ☐☐

SECTION SEVEN - ENDOSCOPY & POST-ENDOSCOPY MANAGEMENT

40. Date & time the endoscopy was performed

☐☐ ☐☐ 2 0 2 2 ☐☐ ☐☐

40a. Were there any delays in arranging an endoscopy related to COVID-19 testing?

☐ Yes ☐ No ☐ Don't Know

41. Where was the endoscopy performed?

☐ Don't know

☐ In the main endoscopy department

☐ Emergency theatre

☐ Critical care

☐ Designated GI bleeding unit

☐ Emergency department

☐ Medical ward

☐ Surgical ward

☐ Other, please state

42. Who was the lead endoscopist for this procedure?

☐ Consultant gastroenterologist

☐ Consultant surgeon

☐ SAS doctor (Associate specialist / Staff grade)

☐ ST / Research fellow / Clinical fellow - supervised

☐ ST / Research fellow / Clinical fellow - unsupervised

☐ Nurse endoscopist

☐ Unknown

43. Did an anaesthetist/intensivist assist with this endoscopy?

☐ Yes *Now answer Q44* ☐ No *Now answer Q46*

44. What was the grade of the most senior anaesthetist?

☐ Consultant

☐ Associate specialist / Staff grade

☐ SpR/ StR / Research fellow / Clinical fellow - supervised

☐ SpR/ StR / Research fellow / Clinical fellow - unsupervised

☐ Unknown

☐ Other

45. Was the patient intubated / general anaesthetic used for this procedure?

☐ Yes *Now answer Q47* ☐ No *Now answer Q46*

46. Which of the following was administered for the endoscopy?

☐ Throat spray (i.e. Lidocaine)

☐ Midazolam

☐ Pethidine

☐ Fentanyl

☐ Flumazenil

☐ Other

☐ None

47. Was there a cause of bleeding found at this endoscopy?

☐ Yes *Now answer Q48* ☐ No *Now answer Q49*

48. What was the endoscopically proven cause of AUGIB? *Select all that apply*

☐ Oesophagitis

☐ Oesophageal ulcer

☐ Gastric ulcer

☐ Duodenal ulcer

☐ Mallory-Weiss tear

☐ Dieulafoy lesion

☐ Oesophageal varices

☐ Gastric varices

☐ Duodenal varices

☐ Portal hypertensive gastropathy

☐ Gastric Antral Vascular Ectasia (GAVE); Talangiectasia

☐ Talangiectasia

☐ Post-sphincterotomy bleed

☐ Other

☐ Upper GI malignancy *Now answer Q48a*

48a. Was the upper GI malignancy…

☐ Oesophageal

☐ Gastric

☐ Duodenal

49. Were there any stigmata of recent haemorrhage recorded at this endoscopy?

☐ Yes *Now answer Q50* ☐ No *Now answer Q51*

50. What were the stigmata? *Select all that apply*

☐ Blood in upper GI tract

☐ Spurting vessel (Forrest 1a)

☐ Oozing blood (1b)

☐ Visible vessel (2a)

☐ Adherent clot (2b)

☐ Nipple sign / Red spot / Wheal markings in varices

51. Were any therapeutic procedures undertaken during this upper GI endoscopy?

☐ Yes *Now answer Q52*   ☐ No *Now answer Q54*

52. What therapeutic procedures were undertaken? *Select all that apply*

☐ BICAP / Heater probe

☐ Endoclip(s) applied

☐ Argon Plasma Coagulation

☐ Haemospray

☐ Endoclot

☐ Purastat

☐ Alternative haemostatic powder/gel

☐ Over the scope clip

☐ Sengstaken tube

☐ Danis stent

☐ Ulcer base injection with adrenaline *Also answer Q52a*

☐ Variceal therapy *Also answer Q52b*

☐ Other, please state

52a. What volume of adrenaline was used in total for this ulcer base injection?

52b. What was the variceal therapy?

☐ Variceal banding

☐ Sclerotherapy

☐ Glue injection

☐ Thrombin injection

53. Did the endoscopist record the outcome of therapeutic procedures on the bleeding?

☐ Yes *Now answer Q53a* ☐ No *Now answer Q54*

53a. Was haemostasis achieved?

☐ Yes ☐ No

54. Did the patient have a biopsy taken for Helicobacter pylori or CLO urease test?
(either at the first or a subsequent endoscopy during admission)

☐ Yes *Now answer Q54a* ☐ No *Now answer Q55* ☐ Don't know *Now answer Q55*

54a. Was the patient positive for Helicobacter pylori?

☐ Yes ☐ No ☐ Don't know

55. Did the endoscopist make a clear plan in the report for: *Select all that apply*

☐ Re-bleeding

☐ Restarting anti-thrombotic agents

☐ Consideration for LMWH

☐ Need for gastroenterology referral

☐ Need for interventional radiology

☐ Need for surgical referral

56. Which of the following were started or continued as treatment for AUGIB after the endoscopy? *Tick all appropriate options and give details of duration of treatment at Q56a-f*

☐ None

☐ PPI Oral

56a. Please provide details of duration of treatment

☐ PPI intravenous boluses

56b. Please provide details of duration of treatment

☐ PPI intravenous infusion

56c. Please provide details of duration of treatment

☐ Terlipressin

56d. Please provide details of duration of treatment

☐ Octreotide

56e. Please provide details of duration of treatment

☐ Antibiotics for UGI bleeding (except H pylori eradication)

56f. Please provide details of duration of treatment

☐ Tranexamic Acid

56g. Please provide details of duration of treatment

57. For patients on antiplatelets, anticoagulants or LMWH, was it restarted post endoscopy?

☐ Yes *Now answer Q57a* ☐ No *Now answer Q58* ☐ Was not discontinued *Now answer Q58*

☐ Was not on antiplatelets *Now answer Q58*

57a. Please indicate which antiplatelets the patient was restarted on, and the date

☐ Aspirin

Date & time it was restarted

				2	0	2	2				

☐ P2Y12 inhibitors *(Clopidogrel, Prasugrel or Ticagrelor)*

Date & time it was restarted

☐☐ ☐☐ 2022 ☐☐ ☐☐

☐ Warfarin

Date & time it was restarted

☐☐ ☐☐ 2022 ☐☐ ☐☐

☐ Direct Oral Anticoagulants (DOACs) *(Apixaban, Rivaroxaban, Edoxaban, Dabigatran)*

Date & time it was restarted

☐☐ ☐☐ 2022 ☐☐ ☐☐

☐ Low Molecular Weight Heparin or unfractionated heparin

Date & time it was restarted

☐☐ ☐☐ 2022 ☐☐ ☐☐

58. Did the patient have evidence of re-bleeding after the endoscopy?

☐ Yes *Now answer Q59 & Q60* ☐ No *Now answer Q61* ☐ Don't know *Now answer Q61*

59. Date when the re-bleeding was noted

☐☐ ☐☐ 2022

60. What was the evidence of re-bleeding? *Select all that apply*

☐ Recurrent hematemesis, coffee ground vomiting or bloody nasogastric aspirate after index endoscopy

☐ Recurrent tachycardia or hypotension after achieving haemodynamic stability

☐ Melena and/or haematochezia following normalisation of stool colour

☐ A reduction in haemoglobin ≥ 2 g/DL after a stable haemoglobin value has been attained

☐ None of the above

61. Was the endoscopy repeated during the admission?

☐ Yes *Now answer Q63* ☐ No *Now answer Q62* ☐ Don’t know *Now answer Q65*

62. Why was the endoscopy not repeated?

☐ Patient was specifically categorised for no active treatment or investigation after the first OGD

☐ Patient self-discharged against medical advice

☐ Patient was transferred / referred for further management (TIPSS/Embolization/Surgery)

☐ Patient died before the OGD could be performed

☐ Repeat endoscopy was not required

Now answer Q65

63. What was the total number of endoscopies for this episode / admission with upper GI bleed?

☐ 2 *Now Answer Q64 and continue to the* ***SECOND ENDOSCOPY*** *section*

☐ 3 or more *Now Answer Q64 and continue to the* ***SECOND ENDOSCOPY*** *section, we will send you additional paperwork to tell us about further endoscopies*

64. What was/were the reason(s) for the repeat procedure? *Select all that apply*

☐ For check/repeat therapeutic procedure

☐ For further bleeding (continued or re-bleeding)

☐ Patient unstable and first procedure had to be abandoned

☐ Insufficient endoscopist expertise at time of first scope

☐ Technical / equipment failure so first unsuccessful

☐ Inadequate views of whole upper GI tract at first endoscopy

☐ Don’t know

SECOND ENDOSCOPY

Endo2: 40. Date & time the endoscopy was performed

				2	0	2	2						

Endo2: 41. Where was the endoscopy performed?

☐ Don’t know

☐ In the main endoscopy department

☐ Emergency theatre

☐ Critical care

☐ Designated GI bleeding unit

☐ Emergency department

☐ Medical ward

☐ Surgical ward

☐ Other, please state

Endo2: 42. Who was the lead endoscopist for this procedure?

☐ Consultant gastroenterologist

☐ Consultant surgeon

☐ SAS doctor (Associate specialist / Staff grade)

☐ ST / Research fellow / Clinical fellow - supervised

☐ ST / Research fellow / Clinical fellow - unsupervised

☐ Nurse endoscopist

☐ Unknown

Endo2: 43. Did an anaesthetist/intensivist assist with this endoscopy?

☐ Yes *Now answer Endo2: Q44* ☐ No *Now answer Endo2: Q46*

Endo2: 44. What was the grade of the most senior anaesthetist?

☐ Consultant

☐ Associate specialist / Staff grade

☐ SpR/ StR / Research fellow / Clinical fellow - supervised

☐ SpR/ StR / Research fellow / Clinical fellow - unsupervised

☐ Unknown

☐ Other

Endo2: 45. Was the patient intubated / general anaesthetic used for this procedure?

☐ Yes *Now answer Endo2: Q47* ☐ No *Now answer Endo2: Q46*

Endo2: 46. Which of the following was administered for the endoscopy?

☐ Throat spray (i.e. Lidocaine)

☐ Midazolam

☐ Pethidine

☐ Fentanyl

☐ Flumazenil

☐ None

☐ Other

Endo2: 47. Was there a cause of bleeding found at this endoscopy?

☐ Yes *Now answer Endo2: Q48* ☐ No *Now answer Endo2: Q49*

Endo2: 48. What was the endoscopically proven cause of AUGIB? *Select all that apply*

☐ Oesophagitis

☐ Oesophageal ulcer

☐ Gastric ulcer

☐ Duodenal ulcer

☐ Mallory-Weiss tear

☐ Dieulafoy lesion

☐ Oesophageal varices

☐ Gastric varices

☐ Duodenal varices

☐ Portal hypertensive gastropathy

☐ Gastric Antral Vascular Ectasia (GAVE); Talangiectasia

☐ Talangiectasia

☐ Post-sphincterotomy bleed

☐ Other

☐ Upper GI malignancy *Now answer Endo2: Q48a*

Endo2: 48a. Was the upper GI malignancy…

☐ Oesophageal

☐ Gastric

☐ Duodenal

Endo2: 49. Were there any stigmata of recent haemorrhage recorded at this endoscopy?

☐ Yes *Now answer Endo2: Q50* ☐ No *Now answer Endo2: Q51*

Endo2: 50. What were the stigmata? *Select all that apply*

- ☐ Blood in upper GI tract
- ☐ Spurting vessel (Forrest 1a)
- ☐ Oozing blood (1b)
- ☐ Visible vessel (2a)
- ☐ Adherent clot (2b)
- ☐ Nipple sign / Red spot / Wheal markings in varices

Endo2: 51. Were any therapeutic procedures undertaken during this upper GI endoscopy?

☐ Yes *Now answer Endo2: Q52* ☐ No *Now answer Endo2: Q54*

Endo2: 52. What therapeutic procedures were undertaken? *Select all that apply*

- ☐ BICAP / Heater probe
- ☐ Endoclip(s) applied
- ☐ Argon Plasma Coagulation
- ☐ Haemospray
- ☐ Endoclot
- ☐ Purastat
- ☐ Alternative haemostatic powder/gel
- ☐ Over the scope clip
- ☐ Sengstaken tube
- ☐ Danis stent
- ☐ Ulcer base injection with adrenaline *Also answer Endo2: Q52a*
- ☐ Variceal therapy *Also answer Endo2: Q52b*
- ☐ Other, please state

Endo2: 52a. What volume of adrenaline was used in total for this ulcer base injection?

Endo2: 52b. What was the variceal therapy?

☐ Variceal banding

☐ Sclerotherapy

☐ Glue injection

☐ Thrombin injection

Endo2: 53. Did the endoscopist record the outcome of therapeutic procedures on the bleeding?

☐ Yes *Now answer Endo2: Q53a* ☐ No *Now answer Endo2: Q54*

Endo2: 53a. Was haemostasis achieved?

☐ Yes ☐ No

Endo2: 54. Did the patient have a biopsy taken for Helicobacter pylori or CLO urease test?
(either at the first or a subsequent endoscopy during admission)

☐ Yes *Now answer Endo2: Q54a* ☐ No *Now answer Endo2: Q55*

☐ Don't know *Now answer Endo2: Q55*

Endo2: 54a. Was the patient positive for Helicobacter pylori?

☐ Yes ☐ No ☐ Don't know

Endo2: 55. Did the endoscopist make a clear plan in the report for: *Select all that apply*

☐ Re-bleeding

☐ Restarting anti-thrombotic agents

☐ Consideration for LMWH

☐ Need for gastroenterology referral

☐ Need for interventional radiology

☐ Need for surgical referral

Endo2: 56. Which of the following were started or continued as treatment for AUGIB after the first endoscopy?

Tick all appropriate options and give details of duration of treatment at Endo2: Q56a-f

☐ None

☐ PPI Oral

Endo2: 56a. Please provide details of duration of treatment

☐ PPI intravenous boluses

Endo2: 56b. Please provide details of duration of treatment

☐ PPI intravenous infusion

Endo2: 56c. Please provide details of duration of treatment

☐ Terlipressin

Endo2: 56d. Please provide details of duration of treatment

☐ Octreotide

Endo2: 56e. Please provide details of duration of treatment

☐ Antibiotics for UGI bleeding (except H pylori eradication)

Endo2: 56f. Please provide details of duration of treatment

☐ Tranexamic Acid

Endo2: 56g. Please provide details of duration of treatment

Endo2: 57. For patients on antiplatelets, anticoagulants or LMWH, was it restarted post endoscopy?

☐ Yes *Now answer Endo2: Q57a* ☐ No

☐ Was not discontinued ☐ Was not on antiplatelets

Endo2: 57a. Please indicate which antiplatelets the patient was restarted on, and the date

☐ Aspirin

Date & time it was restarted

					2	0	2	2					

☐ P2Y12 inhibitors *(Clopidogrel, Prasugrel or Ticagrelor)*

Date & time it was restarted

				2	0	2	2					

☐ Warfarin

Date & time it was restarted

				2	0	2	2					

☐ Direct Oral Anticoagulants (DOACs) *(Apixaban, Rivaroxaban, Edoxaban, Dabigatran)*

Date & time it was restarted

				2	0	2	2					

☐ Low Molecular Weight Heparin or unfractionated heparin

Date & time it was restarted

				2	0	2	2					

Endo2: 58. Did the patient have evidence of re-bleeding after the endoscopy?

☐ Yes *Now answer Endo2: Q59 & Q60* ☐ No *Now answer Q65*

☐ Don't know *Now answer Q65*

Endo2: 59. Date when the re-bleeding was noted

				2	0	2	2

Endo2: 60. What was the evidence of re-bleeding? *Select all that apply*

☐ Recurrent hematemesis, coffee ground vomiting or bloody nasogastric aspirate after index endoscopy

☐ Recurrent tachycardia or hypotension after achieving haemodynamic stability

☐ Melena and/or haematochezia following normalisation of stool colour

☐ A reduction in haemoglobin ≥ 2 g/DL after a stable haemoglobin value has been attained

☐ None of the above

65. Was the patient diagnosed with a variceal bleed?

☐ Yes *Now answer Q66* ☐ No *Now answer Q75*

66. Did the endoscopist feel adequate haemostasis had been achieved?

☐ Yes ☐ No

67. Did the patient have a Sengstaken tube in place at any point during the admission?

☐ Yes *Now answer Q67a* ☐ No *Now answer Q68*

67a. Please tell us about any complications relating to the Sengstaken tube:

☐ No complications

☐ Pressure necrosis

☐ Perforation

68. Does your hospital have a TIPSS service?

☐ Yes *Now answer Q69* ☐ No *Now answer Q68a*

68a. Was the patient discussed for TIPSS with a TIPSS centre prior to hospital discharge?

☐ Yes *Now answer Q68b* ☐ No *Now answer Q70*

68b. Was the patient accepted for TIPSS?

☐ Yes *Now answer Q68c* ☐ No *Now answer Q70*

68c. What was the date and time of transfer?

☐☐ ☐☐ 2 0 2 2 ☐☐ ☐☐ ☐ Unknown

Now answer Q69

69. Did the patient undergo TIPSS?

☐ Yes *Now answer Q69a* ☐ No *Now answer Q70*

69a. What was the date and time of the TIPSS?

☐☐ ☐☐ 2 0 2 2 ☐☐ ☐☐ ☐ Unknown

70. Was the patient discharged on non-selective beta blockers?

☐ Yes *Now answer Q70a* ☐ No *Now answer Q71*

70a. Tell us about the drug and dose:

☐ Carvedilol

Dose (mg) ☐ Frequency ☐ OD ☐ BD

☐ Propranolol

Dose (mg) ☐ Frequency ☐ OD ☐ BD

☐ Nadolol

Dose (mg) ☐ Frequency ☐ OD ☐ BD

71. Was a repeat scope planned at time of discharge?

☐ Yes *Now answer Q71a* ☐ No *Now answer Q75*

71a. Duration in which planned to be repeated:

☐ weeks

The following section is only for those patients who did not have an OGD during admission

72. Please tell us why an inpatient OGD was not performed:

- ☐ An inpatient OGD was not indicated clinically
- ☐ This patient was specifically categorised for no active treatment or investigation when they first presented with AUGIB
- ☐ The patient self-discharged before the OGD could be performed
- ☐ The patient was transferred to another hospital for further management
- ☐ The patient died before the OGD could be performed.

73. Was the patient discharged without a plan for endoscopy?

☐ Yes ☐ No ☐ Not applicable

74. Was an endoscopy requested to be done as an outpatient?

☐ Yes ☐ No ☐ Not applicable

SECTION EIGHT - RADIOLOGICAL INTERVENTION

75. Did the patient have radiological intervention for the upper GI bleed?

☐ Yes *Now answer Q76* ☐ No *Now answer Q83*

76. What was the date and time of the interventional radiology?

☐☐ ☐☐ 2 0 2 2 ☐☐ ☐☐

77. Which procedure did the patient undergo? *Select all that apply*

☐ Diagnostic angiography alone

☐ Diagnostic and therapeutic angiography *Now provide further details below*

Which vessel/branch was therapeutically embolized?

What was used for the embolization? *Select all that apply*

☐ Coils

☐ Plugs

☐ Gelfoam

☐ Particles

☐ Glue

☐ Onyx or equivalent

☐ Other, please state

☐ Empiric embolization undertaken because there was no identified bleeding on angiography but bleeding was seen on CT angiography. *Now provide further details below*

Which vessel/branch was empirically embolized?

What was used for the embolization? *Select all that apply*

- ☐ Coils
- ☐ Plugs
- ☐ Gelfoam
- ☐ Particles
- ☐ Glue
- ☐ Onyx or equivalent
- ☐ Other, please state

☐ Empiric embolization undertaken because there was no identified bleeding on angiography but bleeding was seen on prior endoscopy *Now provide further details below*

Which vessel/branch was empirically embolized?

What was used for the embolization? *Select all that apply*

☐ Coils

☐ Plugs

☐ Gelfoam

☐ Particles

☐ Glue

☐ Onyx or equivalent

☐ Other, please state

☐ Empiric embolization undertaken because there was no identified bleeding on angiography or on prior endoscopy. *Now provide further details below*

Which vessel/branch was empirically embolized?

What was used for the embolization? *Select all that apply*

- ☐ Coils
- ☐ Plugs
- ☐ Gelfoam
- ☐ Particles
- ☐ Glue
- ☐ Onyx or equivalent
- ☐ Other, please state

☐ Transjugular Intrahepatic Portosystemic Shunt (TIPSS) only

☐ Transjugular Intrahepatic Portosystemic Shunt (TIPSS) with embolization performed at time of procedure *Now provide further details below*

What was used for the embolization? *Select all that apply*

- ☐ Coils
- ☐ Plugs
- ☐ Gelfoam
- ☐ Particles
- ☐ Glue
- ☐ Onyx or equivalent
- ☐ Other, please state

☐ Transjugular Intrahepatic Portosystemic Shunt (TIPSS) with delayed Embolization. *Now provide further details below*

What was used for the embolization? *Select all that apply*

- ☐ Coils
- ☐ Plugs
- ☐ Gelfoam
- ☐ Particles
- ☐ Glue
- ☐ Onyx or equivalent
- ☐ Other, please state

☐ Balloon-occluded Retrograde Transvenous Obliteration (BRTO)

☐ Plug-assisted Retrograde Transvenous Obliteration (PARTO) or coil-assisted Retrograde Transvenous Obliteration (CARTO) *Now provide further details below*

What was used for the PARTO or CARTO? *Select all that apply*

☐ Coils

☐ Plugs

☐ Gelfoam

☐ Particles

☐ Glue

☐ Onyx or equivalent

☐ Other, please state

78. Please tell us about any other treatments: *(i.e. spasmodics, etc.)*

79. Was the bleeding controlled?

☐ Yes ☐ No ☐ Don't know

80. Did the patient have evidence of re-bleeding after the first procedure?

☐ Yes *Now answer Q80a-82* ☐ No ☐ Don't know

80a. Date when the re-bleeding was noted

☐☐ ☐☐ 2 0 2 2

81. Was re-embolization attempted?

☐ Yes *Now answer Q82* ☐ No ☐ Don't know

82. Was the repeat IR procedure successful in controlling the GI bleeding?

☐ Yes ☐ No

SECTION NINE - SURGICAL INTERVENTION

83. Did the patient have surgery to control the GI bleeding?

☐ Yes *Now answer Q84* ☐ No *Now answer Q91*

84. What was the date and time of the surgery?

						2	0	2	2								

85. What was the ASA status of the patient at the time of surgery?

☐ I

☐ II

☐ III

☐ IV

☐ Not documented

86. What was the reason for surgery? *Select all that apply*

☐ For further uncontrolled bleeding

☐ Stigmata of recent haemorrhage / high risk

☐ For malignancy

☐ For peritonitis / perforation

☐ For complications related to endoscopy

☐ For complications related to interventional radiology

☐ Other, please state

87. Please state the type of surgery

☐ Open

☐ Laparoscopic

88. What was the procedure?

☐ Duodenotomy and underrunning of the vessel

☐ Pyloro-duodenotomy and underrunning of the vessel

☐ Oversew or plication of the ulcer

☐ Excision of the ulcer with vagotomy and pyloroplasty

☐ Gastrectomy (partial or other)

☐ Under running of gastric ulcer + biopsies

☐ + / - Ligation of gastroduodenal artery

☐ Laparoscopic / Open wedge excision of bleeding lesion

☐ Other comments

89. What was the grade of the operating surgeon?

☐ Consultant surgeon

☐ Associate specialist/staff grade

☐ SpR/StR/research fellow/clinical fellow - supervised

☐ SpR/StR/research fellow/clinical fellow - unsupervised

☐ Unknown

☐ Other

90. What was the grade of the most senior anaesthetist?

☐ Consultant

☐ Associate specialist / Staff grade

☐ SpR/ StR / Research fellow / Clinical fellow - supervised

☐ SpR/ StR / Research fellow / Clinical fellow - unsupervised

☐ Unknown

☐ Other

SECTION TEN - OVERALL TRANSFUSION SUMMARY Please complete for all patients

91. Did the patient receive red blood cell transfusion(s) at any time for the AUGIB?

☐ Yes *Please also complete Table 5 at the end of this questionnaire* ☐ No

92. Did the patient receive platelet transfusion(s) at any time for the AUGIB?

☐ Yes *Please also complete Table 6 at the end of this questionnaire* ☐ No

93. Did the patient receive Fresh, Frozen Plasma transfusion(s) at any time for the AUGIB?

☐ Yes *Please also complete Table 7 at the end of this questionnaire* ☐ No

94. Did the patient receive prothrombin complex concentrate infusion(s) at any time for the AUGIB?

☐ Yes *Please also complete Table 8 at the end of this questionnaire* ☐ No

95. Did the patient receive fibrinogen infusion(s) at any time for the AUGIB?

☐ Yes *Please also complete Table 9 at the end of this questionnaire* ☐ No

96. Did the patient receive cryoprecipitate transfusion(s) at any time for the AUGIB?

☐ Yes *Please also complete Table 10 at the end of this questionnaire* ☐ No

97. Did the patient receive Human Albumin Solution (HAS) at any time for the AUGIB?

☐ Yes *Please also complete Table 11 at the end of this questionnaire* ☐ No

98. Did the patient receive reversal agents for DOACS at any time for the AUGIB?

☐ Yes *Now answer Q98a* ☐ No

98a. Please tell us the name and dose of the agent given

SECTION ELEVEN - OVERALL CLINICAL SUMMARY

99. Was there documentation on ceiling of care?

☐ Yes *Now answer Q99a & b* ☐ No *Now answer Q100*

99a. What was the date and time (if available) of the documentation?

				2	0	2	2						

99b. What was the ceiling of care?

☐ Ward based

☐ Organ support but not for intubation and invasive ventilation

☐ For full escalation

100. Was there a DNACPR documentation?

☐ Yes *Now answer Q100a* ☐ No *Now answer Q101*

100a. DNACPR form?

☐ Yes *Now answer Q100b* ☐ No *Now answer Q101*

100b. What was the date and time (if available)?

				2	0	2	2						

101. Which one of the following statements best summarises the outcome of the AUGIB episode for this patient? *Please select only ONE option*

- ☐ AUGIB not requiring endoscopy
- ☐ AUGIB in a patient not for active investigation/therapy
- ☐ AUGIB requiring diagnostic endoscopy only
- ☐ AUGIB requiring therapeutic endoscopy to control bleeding
- ☐ AUGIB requiring surgery or radiological intervention to control bleeding
- ☐ Self-discharge against medical advice before endoscopic intervention
- ☐ Death during admission from AUGIB
- ☐ Death during admission from another cause

102. Did the patient die during the admission for AUGIB?

☐ Yes *Now answer Q102a* ☐ No *Now answer Q105*

102a. What was the date and time of death?

				2	0	2	2				

103. Is there a record of the patient's cause of death?

☐ Yes *Now answer Q104* ☐ No *Now complete tables 1-11, as appropriate*

104. Please tell us about the cause of death

1a	
1b	
1c	
2	

105. Is the patient still in hospital more than 28 days after first presenting with AUGIB?

☐ Yes *Now complete tables 1-11, as appropriate* ☐ No *Now answer Q106* ☐ Don't know

106. What was the date of discharge?

				2	0	2	2

107. Did the patient get re-admitted for another episode of AUGIB within 28 days of being discharged?

☐ Yes *Now answer Q107a* ☐ No ☐ Don't know

107a. What was the date of re-admission?

				2	0	2	2

Now complete tables 1-11, as appropriate

Table 1 - Full Blood Count tests

	Date of test result	Time of test result (if available)	Hb value (g/l)	Platelet count x109/L
**FBC1**<br>First FBC recorded after presentation with AUGIB *(at time of admission - if admitted with GI bleed; at time of bleeding – in patient hospitalised for other reasons)*				
**FBC2**<br>Lowest recorded haemoglobin during admission/ episode of AUGIB				
**FBC3**<br>Lowest recorded platelet count during admission/ episode of AUGIB				
**FBC3**<br>Last recorded FBC prior to discharge / death / Transfer during admission/ episode of AUGIB				

Table 2 - Clotting Screen Tests

	Date of test result	Time of test result (if available)	INR (if Available)	Prothrombin time (Secs)	Control Values (Secs)	Fibrinogen level (if available)
**CS1**<br>First INR / PT during admission / AUGIB episode *(at time of admission - if admitted with GI bleed; at time of bleeding – in patient hospitalised for other reasons)*						
**CS2**<br>Highest INR / PT during admission / AUGIB episode						

Table 3 - Biochemistry profile (BCP)

	**Date of test result**	**Time of test result (if available)**	**Urea (mmol/L)**	**Creatinine (µmol/L)**	**Bilirubin (µmol/L)**	**Albumin (g/L)**	**Na+ (mmol/L)**
**BCP1** First biochemistry recorded after presentation with AUGIB *(at time of admission - if admitted with GI bleed; at time of bleeding – in patient hospitalised for other reasons)*							

Table 4 – Blood lactate level

	**Date of test result**	**Time of test result (if available)**	**Blood lactate (mmol/L)**
Peak blood lactate recorded prior to endoscopy after onset of AUGIB (if available)			

Table 5 - RED CELL Transfusion Episodes *(All red blood cells consecutively transfused within a 24-hour period)*

	**Date of Transfusion**	**Indication**<br>*1. Acute bleeding*<br>*2. Anaemia in a patient with acute coronary syndrome*<br>*3. Anaemia*	**No. of units transfused**	**Pre- transfusion Hb (g/L)**	**Date of test**	**Time of test**	**Post-transfusion Hb (g/L)**	**Date of test**	**Time of test**
RBC1									
RBC2									
RBC3									
RBC4									
RBC5									
RBC6									
RBC7									
RBC8									
RBC9									
RBC10									

Table 6 - Platelets Transfusion Episodes *(All platelets consecutively transfused within a 24-hour period)*

	**Date of transfusion**	**Indication**<br>*1. Massive haemorrhage*<br>*2. PLT count ≤ 50 x 10^9/l pre-procedure*<br>*3. Other (provide specific clinical details)*	**No. of pools of platelets transfused**	**Pre-transfusion platelet count (x 10^9)**	**Date of test**	**Time of test**	**Post-transfusion platelet count (x 10^9)**	**Date of test**	**Time of test**
PLT1									
PLT2									
PLT3									
PLT4									
PLT5									
PLT6									
PLT7									
PLT8									
PLT9									
PLT10									

Table 7 - Fresh Frozen Plasma (FFP)Transfusion Episodes *(All FFP consecutively transfused within a 24-hour period)*

	**Date of Transfusion**	**Indication**<br>*1. Massive haemorrhage*<br>*2. INR ≥ 1.6 with bleeding*<br>*3. INR ≥ 1.6 and pre-procedure*<br>*4. Other (provide specific clinical details)*	**No. of units of FFP transfused**	**Pre-transfusion INR/PT (PT in seconds)**	**Date of test**	**Time of test**	**Post-transfusion INR/PT after transfusion**	**Date of test**	**Time of test**
FFP1									
FFP2									
FFP3									
FFP4									
FFP5									
FFP6									
FFP7									
FFP8									
FFP9									
FFP10									

Table 8 - Prothrombin Complex Concentrate (PCC) Infusion Episodes *(All PCC consecutively infused within a 24-hour period)*

	**Date of infusion**	**Indication**<br>*1. Correction of warfarin anticoagulant effect*<br>*2. Other (provide specific clinical details)*	**Dose of PCC (IU) infused**	**Pre-infusion INR/PT (PT in seconds)**	**Date of test**	**Time of test**	**Post-infusion INR/PT (PT in seconds)**	**Date of test**	**Time of test**
PCC1									
PCC2									
PCC3									
PCC4									
PCC5									

Table 9 - Fibrinogen Infusion Episodes *(All fibrinogen consecutively infused within a 24-hour period)*

	**Date of Infusion**	**Indication**<br>1. *Massive haemorrhage*<br>2. *Fibrinogen ≤ 1.0g/l & pre-procedure*<br>3. *Other (provide specific clinical details)*	**Dose of fibrinogen (mg) infused**	**Pre-infusion fibrinogen**	**Date of test**	**Time of test**	**Post-infusion fibrinogen**	**Date of test**	**Time of test**
FIB1									
FIB2									
FIB3									
FIB4									
FIB5									

Table 10 - Cryoprecipitate Transfusion Episodes *(All cryoprecipitate consecutively transfused within a 24-hour period)*

	**Date of Transfusion**	**Indication**<br>*1. Massive haemorrhage*<br>*2. Fibrinogen ≤ 1.5g/l & clinically significant bleeding*<br>*3. Fibrinogen ≤ 1.0g/l & pre-invasive procedure/surgery with risk of significant bleeding*<br>*4. Other (provide specific clinical details)*	**No. of pooled units of cryoprecipitate transfused**	**Pre-transfusion fibrinogen**	**Date of test**	**Time of test**	**Post-transfusion fibrinogen**	**Date of test**	**Time of test**
Cryo1									
Cryo2									
Cryo3									
Cryo4									
Cryo5									

Table 11 - Human Albumin Solution Infusion Episodes *(All HAS consecutively infused within a 24-hour period)*

	**Date of infusion**	**No. of bottles of 4.5% human albumin infused**	**No of bottles of 20% human albumin infused**
HAS1			
HAS2			
HAS3			
HAS4			
HAS5			
HAS6			
HAS7			
HAS8			
HAS9			
HAS10			
