# Supplementary material for: Acute upper gastrointestinal bleeding in the UK: 2022 audit update
Source: Gut. 2025 Nov 19;75(4):e335134. doi: 10.1136/gutjnl-2025-335134 (PMC13018800; doi:10.1136/gutjnl-2025-335134)
Supplement: online supplemental file 3 [file gutjnl-75-4-s003.pptx]

## Slide 1
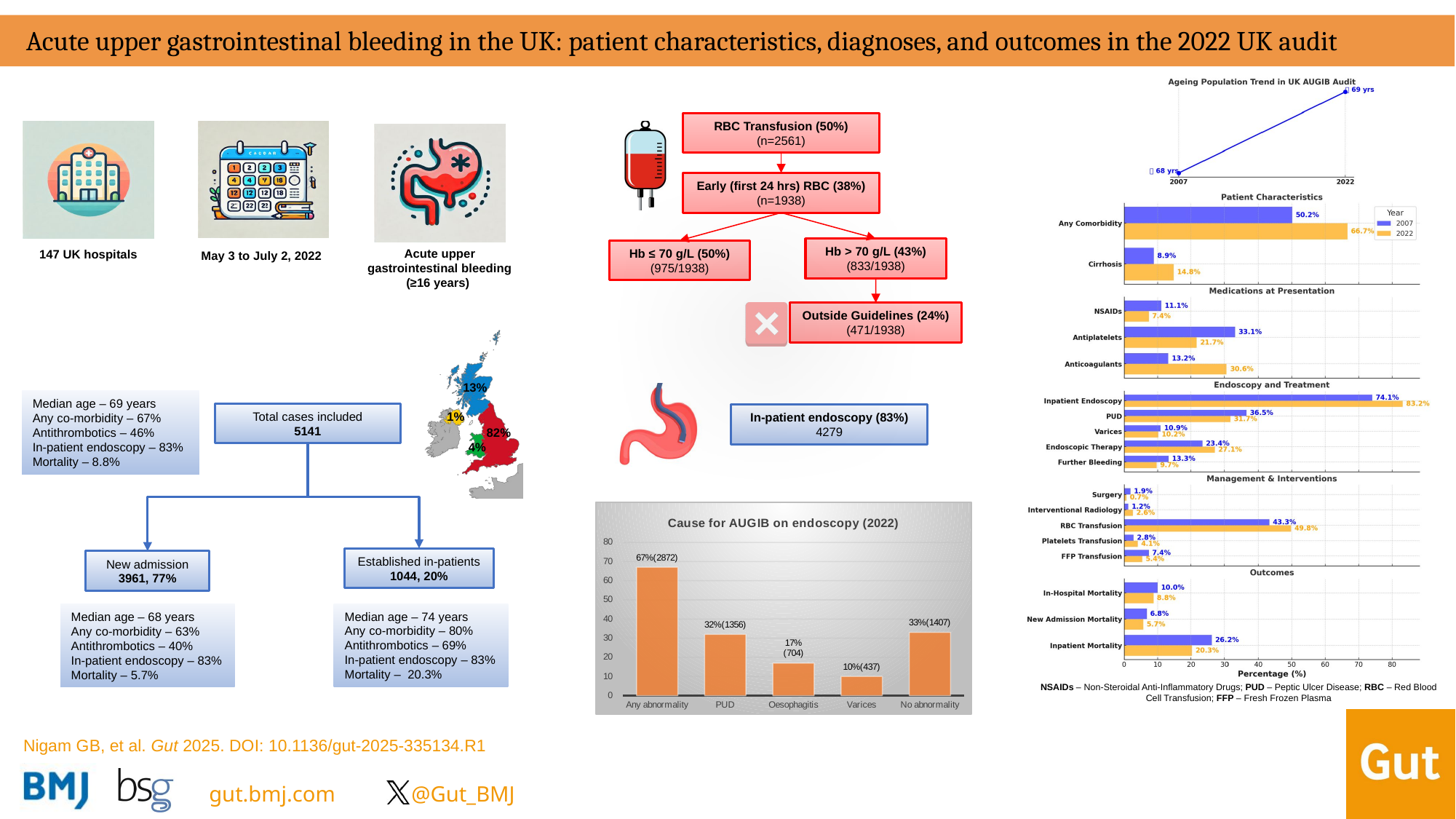

Acute upper gastrointestinal bleeding in the UK: patient characteristics, diagnoses, and outcomes in the 2022 UK audit
RBC Transfusion (50%)
(n=2561)
Early (first 24 hrs) RBC (38%)
(n=1938)
Hb > 70 g/L (43%)
(833/1938)
Acute upper gastrointestinal bleeding (≥16 years)
Hb ≤ 70 g/L (50%)
(975/1938)
147 UK hospitals
May 3 to July 2, 2022
Outside Guidelines (24%)
(471/1938)
13%
Median age – 69 years
Any co-morbidity – 67%
Antithrombotics – 46%
In-patient endoscopy – 83%
Mortality – 8.8%
Total cases included
5141
1%
In-patient endoscopy (83%)
4279
82%
4%
### Chart: Cause for AUGIB on endoscopy (2022)
| Category | First 24 hrs |
|---|---|
| Any abnormality | 67.0 |
| PUD | 32.0 |
| Oesophagitis | 17.0 |
| Varices | 10.0 |
| No abnormality | 33.0 |Established in-patients
1044, 20%
New admission
3961, 77%
Median age – 74 years
Any co-morbidity – 80%
Antithrombotics – 69%
In-patient endoscopy – 83%
Mortality – 20.3%
Median age – 68 years
Any co-morbidity – 63%
Antithrombotics – 40%
In-patient endoscopy – 83%
Mortality – 5.7%
NSAIDs – Non-Steroidal Anti-Inflammatory Drugs; PUD – Peptic Ulcer Disease; RBC – Red Blood Cell Transfusion; FFP – Fresh Frozen Plasma
Nigam GB, et al. Gut 2025. DOI: 10.1136/gut-2025-335134.R1
gut.bmj.com
@Gut_BMJ
