# Supplementary material for: Acute upper gastrointestinal bleeding in the UK: 2022 audit update
Source: Gut. 2025 Nov 19;75(4):e335134. doi: 10.1136/gutjnl-2025-335134 (PMC13018800; doi:10.1136/gutjnl-2025-335134)
Supplement: online supplemental file 4 [file gutjnl-75-4-s004.docx]

**Aberdeen Royal Infirmary**

Lindsay McLeman

Maciej Adler

**Addenbrooke's Hospital**

Gareth Corbett

Ahmed Feroz

Abdul Hameed Rahini

Ahmad Hassan

Catherine Katabira

Dunecan Massey

Madilia Muhammad

Farooq Mirza

Mohammad Choudhury

Nyo Lai Yee Win

Syad Ali Bilal Hassan

**Aintree University Hospital**

Neil Kapoor

Doug Penman

**Altnagelvin Area Hospital**

Charles Ferguson

Ciaran Francis Magee

**Arrowe Park Hospital**

Adrian Thuraisingham

Nikki Summers

Ioannis Papamargaritis

James Colclough

Joseph Parsons

**Barnsley Hospital**

Elmuhtady Said

Thomas Archer

Alex Calderbank

Elmuhtady Said

Ali Mahdi

Imran Ahmad

Kimberley Monks

Martha Ellis

Matt Hughes

Raaid Jamil

Rusyai Zalynda

**Basingstoke & North Hampshire Hospital**

Mike Reynolds

Chinonso Nwoguh

**Bedford Hospital**

Jacqelyn Harvey

Sim Yee Lim

**Birmingham Heartlands Hospital**

Muhammad Azhar Hussain

Hurooul Aain

Krithi Shamanur

Nahid Hassan

Preethi George Pandeth

**Borders General**

Jonathan Fletcher

Daniel Lynch

Amir Khan

**Bradford Royal Infirmary**

Sulleman Moreea

Sarah Jowett

Imran Iqbal

Jade Palmer

Kameel Khan

Mahmoud Bakr

Paramdeep Duggal

Sonia Moteea

Vandana Ruggoo

**Bristol Royal Infirmary**

Jonathan Tyrell-Price

Hannah Donnelly

Lydia Neuberger

Molly Flint

**Caithness General Hospital**

Marcin Szczepanski

Nils Fritsch

**Causeway Hospital**

Gaurav Manikpure

Edvard Volcek

Ajeet Kumar

Amrita Gurung

Gaurav Manikpure

Melissa Flynn

Olivia McConaghie

Phelim McPolin

Rajesh Veetil

**Charing Cross Hospital**

Anet Soubieres

Aaron Bhakta

**Chelsea & Westminster Hospital**

Neerav Joshi

Adham Chakhachiro

Dev Chatterjee

Emer Kilbride

Golnoush Seyedzenouzi

Pyei Aung

Richard Hackett

Utkarsh Ojha

Michael Carbonell

**Chesterfield Royal**

Keith Dear

Mohsen Eldragini

**Colchester Hospital**

Ian Gooding

Theo Panagaris

Aye Phyo

**Countess of Chester Hospital**

Tristan Townsend

Junaid Akhtar

Craig Wyatt

Parmilan Gill

Reea Khanna

Samir Sulaiman

Tristan Townsend

**Croydon University Hospital**

Sanjay Gupta

Michael Colwill

**Cumberland Infirmary**

Sherif Shabana

Mohamed Osman

Deborah Gibson

Paul McClymont

Sherif Shabana

**Darent Valley Hospital**

Nicola Grasso

Sun Mi Ha

Zeeshan Raiput

**Darlington Memorial Hospital**

Anjan Dhar

Danielle Rayner

**Derriford Hospital**

Fahd Baqai

Mohamed Waddah

Madina Mohamed

Mutaz Taha

Sabria Islam

Syed Aaquil Hasan Syed Javid Hasan

**Doncaster Royal Infirmary**

Anthony Chappell

Abuajela Sreh

Chinyere Ochuba

Connor Cotton

Corrie Bowers

Daniel Camlfield

Maaz Nayyer

Matthew Taylor

Moaz Ahmad

Mohamed Ramadan

Sandip Samanta

Sarah Anderson

Thomas Lovering

**Dorset County Hospital**

James Shutt

Liz Bradbury

**Dumfries & Galloway Royal Infirmary**

Mathis Heydtmann

Moawad Mikayed Mohamed

Abdelkader Mahgoub

**Ealing Hospital**

Sohail Shariq

Krishna Shah

Anna Marfin

Jessica Padley

Zahra Mohamedali

**East Surrey Hospital**

Matthew Cowan

Shi Jie Looi

**Forth Valley Royal Hospital**

Joanna Leithead

**Frimley Park Hospital**

Thomas Shepherd

Imogen Sutherland

Elliott Taylor

Barath Baiju

Emily Cooper

Emir Lacevie

Hala El Tahir

Kelan Pascoe

Layla Ganjian

Rebecca Jurdon

**Furness General Hospital**

John Keating

Ahmed Hamdy

**George Eliot Hospital**

Edmond Sung

Walid Mohammed Mujib Choudhary

**Glan Clwyd Hospital**

Aram Baghomian

Hamza Abdelrahim

Asad Baig

**Glasgow Royal Infirmary**

Adrian Stanley

Josh Palmer

**Gloucester Royal Hospital**

Coral Hollywood

Elinor Littlewood

Robbie Adamson

Sophie James

**Good Hope Hospital**

Mujeeb Makki

**Harrogate District Hospital**

Jon Harrison

Hannah Wynn

**Hillingdon Hospital**

Arun Rajendran

Charlotte Skinner

**Hinchingbrooke Hospital**

Anita Gibbons

Krithivasan Praman

Babangida Iliyasu Haruna

Chisom Nwanejuafor

Suhair Ashiq Ali

**Homerton Hospital**

Laura Marelli

Nora Thoa

**Huddersfield Royal Infirmary**

Simon Gonsalves

Puneet Chhabra

Ahmed Rajab

Anuj Gandagule

Angela Matijevic

Ghalia Alia

Hunny Khurana

Jamal Al-Yousofi

Jeanne Babol

Maha Ejaz

Ndidamaka Offor

Shaista Hussain

Sion Roberts

Sophie Price

Sylvia Kinstler

**Hull Royal Infirmary**

Anca Staicu

**Ipswich Hospital**

Hemant Laxaman

Obinna Onwuteaka

**James Paget University Hospital**

Rawya Badreldin

Zeshan Choudry

**John Radcliffe Hospital**

Adam Bailey

Charis Manganis

Archie Lodge

Gaurav Nigam

Julia Pakpoor

Kitty Phillips

Mae Eales

Mo Dada

Solange Bramer

Caitlin Benham

Catherine Seymour

Catriona Phillips

Tabitha Gould

**Kettering General Hospital**

Amr Eldahshan

Solange Serna

Ghayyur Khalil

Haider Mirza

**King's College Hospital**

Debbie Shawcross

Hermon Amanuel

Abdul Samad

Fatima Shahid

M Mohamed

Saira Siddiqui

**King’s Mill Hospital**

Stephen Foley

Mostafa Sherif el-Gindy

**Kingston Hospital**

Ralph Greaves

Ralph Greaves

Rachel Edwards

**Leicester Royal Infirmary**

Aye Aye Thi

Mohamed Shiha

**Leighton Hospital**

Naveen Mohandas

Joshua Muir

**Lincoln County Hospital**

Sharon Sinha

**Lister Hospital**

James Evans

Douglas Corrigall

Mohamed Elseragy

**Luton & Dunstable University Hospital**

Sophie Sinclair

**Manor Hospital Walsall**

Amanda Jane Hughes

Aniruddha Jog & Asif Yasin

Mehrab Rasheed

**Medway Maritime Hospital**

Gabor Sipas

Muneer Abbas

Hsuyadanar Aung

Khine-Zan Wai

Kulprasad Chongbang

Mohamed Ghanem

Pooja Devi

**Milton Keynes General Hospital**

Ravi Madhotra

Arjun Prakash

Adeel Ahmad

Carmen Vlase

Martha Murdoch

Sania Mushtaq

Mohammed Shaheer

Pandara Arakkal

**Morriston Hospital**

Umakant Dave

Mesbah Rahman

**Musgrove Park Hospital**

Emma Wesley

Christina Owen

**New Cross Hospital**

Andrew Veitch

Raheel Anjum

**Newham University Hospital**

Vasu Kulhalli

Swapnil Khose

**Ninewells Hospital**

Michael Miller

Peter Cartlidge

**Norfolk & Norwich University Hospital**

Andrew Douds

Rahim Khan

Jessica Wong

John Thomas

Mie Thu Ko

Usama Aslam

**North Middlesex University Hospital**

Debasis Majumdar

Lynn Affarah

**Northampton General Hospital**

Titus Thomas

Rohan Tariq

Mansur Mohammed

Sarath Kumar

**Northumbria Specialist Emergency Care Hospital**

Tom Lee

**Northwick Park Hospital**

Adam Haycock

Ali Al-Adhami

**Pinderfields Hospital**

Andrea Nicholls

Lewis Germain

B Naw R Aung Din

Majd Abusharar

Sam Murray

**Princess Alexandra Hospital**

Mahmoud Ahmed Elsaid Elkaramany

Federica Merlini

Albert Egwele

Annette Nethersole

Jie Tong

Khadija Stone

**Prince of Wales Hospital**

Clement Lai

Huw Thomas

**Queen Alexandra Hospital**

Pradeep Bhandari

**Queen Elizabeth Hospital Birmingham**

Efe Ejenavi

Athesham Zafar

George Howell

Mehereen Murshed

Muhammad Javaid Iqbal

**Queen Elizabeth Hospital Gateshead**

Raheel Qureshi

**Queen Elizabeth Hospital Greenwich**

Aathavaan Loganayagam

Rawan Al Soud

Brooke Smart

Charlotte Eden

James Dunn

Jonathan Curtis

Nitya Matcha

**Queen's Hospital Burton**

Riaz Dor

**Queen's Medical Centre**

Martin James

Abhishek Sheth

Kristian Wild

Luis Machado

Martin James

Peter Eddowes

Samuel Dilks

Utkarsha Basu

**Raigmore Hospital**

Alan Grant

Hamish Myers

**Royal Alexandra Hospital Paisley**

Inamul Mulhaq

**Royal Berkshire Hospital**

Nishay Chandra

Kharishma Dhera

Chirag Gadhia

Farooq Chaudhary

James Kennedy

Jennifer Kent

Udani Mahamithawa

ZawMyo Aung

**Royal Bolton Hospital**

Nick Wang

Katharine Teasdale

Isabella Girling

Peter McMahon

Kimberley Butler

Shabbir Jivanjee

**Royal Bournemouth Hospital**

Jo Tod

Muhammad Asad

Hassan Sherif

Liam Evans

Sian Meldrum

**Royal Cornwall Hospital**

Keith Sau

Joel James

Arran Williamson

Cisel Boyuegri

Eimon Khine

Joy Worthington

Khine Thu

Mohammad Ghannam

Monica Andrawes

**Royal Derby Hospital**

Said Din

Muhammad Nasim

Emily Tucker

Faisal Baig

Islam Mubashwirul

Mohammed AlShawwaf

Rachel Lai

**Royal Free Hospital**

Jonathan Potts

Alexander Hung

**Royal Glamorgan Hospital**

David Samuel

Darrien Henry

Aneet Kumar

Ben Bridgewater

Ben Pyrke

David Purchase

Eugene Er

Lilian Lau

Richard Vaughan

Rosie McDonald

**Royal Hampshire County Hospital**

Corrine Brooks

Melissa Zhao

**Royal Infirmary of Edinburgh**

Nick Church

Paul Brennan

**Royal Lancaster Infirmary**

John Keating

Julia Moradi

**Royal Liverpool University Hospital**

Andrew Moore

Michelle Sherwin

**Royal Oldham Hospital**

Anirudh Bhandare

Akshay Juwarkar
Eleanor Liu
Nzubechukwu Ozokwelu

**Royal Preston Hospital**

Michael Finegan

Khurram Bin Raees

Lara Satter

Muhammad Aneeb Sabir

Rayhan Gasiea

Shazaib Shahzad

**Royal Shrewsbury Hospital**

Mohamed Mohyeldin Mahgoub

Wail Mostafa

**Royal Surrey County Hospital**

Kallilopi Alexandropoulou

Abhishek Ray

Conor McManaman

Giriraj Raderam

Henry Eynon-Lewis

Khai Leow

Maja Kaladjiska

**Royal United Hospital**

John Saunders

Laura Backhouse

**Royal Victoria Hospital Belfast**

Inder Maine

Andrew Spence

**Royal Victoria Infirmary
Newcastle**

Chris Mountford

Jamie Catlow

**Salford Royal Hospital**

Clare Omerod

Andrew Wong

**Salisbury District Hospital**

Ali Samar

Prashant Dwivedi

Amir Liaqat

Lujan Hassan

Maryam Nasim

**Sandwell District General Hospital**

Imran Ghanghro

**Sheffield Teaching Hospitals**

Alex Ball

Victoria Knott

Al-Hassan Ghodief

Emily Fenner

Jo Buck

John Finnen

Khubaib Malik

Lizzie Peat

Mollie Canavan

Rusyai Ramli

Scarlett Strickland

Thiri Myat

Uzma Asraf

Waleed Ahmed

**South Tyneside District Hospital**

Rohit Sinha

Sarah Manning

**Southampton General Hospital**

Nadeem Tehami

Patricia Duarte

Faisal Nawaz

**Southend University Hospital**

Sharoz Rabbani

Amal Najdawi

Ioannis Koumoutsos

Manal Mamoun

Mehul Amin

Muhammad Khan

Namg Ngin Hom

Rifat Ershad

Win Lae Lae Aung

Wint Wah Oo

Sarala Janarthan

**Southmead Hospital**

Zeino Zeino

Claire Hannon

Jacqueline Roy

**Southport & Formby District General Hospital**

Mike Roberts

**St. George's University Hospital**

Jamal Hayat

Gareth Sadler

Basil Ahmad

Joseph Cooney

**St. James University Hospital**

Ruchit Sood

Kalyan Peddada

Arif Atique

Ben Wildgoose

Hicham Daadaa

Mahmud Elomrani

Muhammad Taha Khan

Yaseer Khan

Ethar Abd Al Shakour

**St. Mary's Isle of Wight**

Julie Parrack

**St. Mary's Paddington**

Lakshmana Ayaru

Stephanie Poo

Anushkumar Vasireddy

Joanna Meng

Jodie Russell

Lakshmana Ayaru

Madelaine Graydon

Thomas Rassam

Varun Nadkarni

Woon Senn Koh

Yuri Im

**St. Thomas' Hospital**

Jason Dunn

Mandour Omer

**Stoke Mandeville Hospital**

David Gorard

Mohamed Ibrahim

**Sunderland Royal Hospital**

Rohit Sinha

Khurum Kakeem

Dominic Maxfield

Alastair Coulson

Lois McMaster

**The Grange University Hospital**

Rhodri Davies

Don Edward Rangedara

**The Great Western Hospital**

Manish Hegde

Rebecca Anderson

**The Horton General Hospital**

Rebecca Palmer

Jessiya Veliyankodan Parambil

Jenny Tempest-Mitchell

Angad Ryatt

**The James Cook University Hospital**

John Greenaway

Alaa Mohamed Ali

**The Queen Elizabeth Hospital King's Lynn**

Shailesh Karanth

**The Queen Elizabeth University Hospital Glasgow**

Jude Morris

Emily Brownson

**The Royal London Hospital**

Patrick Wilson

Josh McGuire

Hal Brindley

Sean Carlson

Sungjae Hwang

Maria Aslam

Mehul Patel

Michael James

Estefania Moreno

**The Ulster Hospital**

Tony Tham

Rebecca O'Kane

**The Whittington Hospital**

Sheena Mankodi

Clive Onnie

**The York Hospital**

Prashant Kant

Najeeb Ullah Khan

**Torbay Hospital**

James Neale

Amin Abdulgader

**University College Hospital**

Mohamed Hussein

**University Hospital Coventry**

Ben Disney

Katherine Arndtz

**University Hospital Crosshouse**

Kevin Robertson

Caroline McCloskey

**University Hospital Llandough**

Hasan Haboubi

**University Hospital North Durham**

Deepak Kejariwal

Danielle Rayner

**University Hospital North Tees**

Iosif Beintaris

Darikha Senanayake

**University Hospital Wishaw**

Marc Cram

Alexander Grayston

**Victoria Hospital Kirkcaldy**

Katharine Pollock

Vaishali Ranade

Lucy Arrowsmith

Naomi Gunn

**Warrington & Halton Hospital**

Sundaramoorthy Bharathi

Anish John Kuriakose Kuzhiyanjal

Charlotte Eaton-Hart

Divya Bhimireddy

Sabrina Pamela Sookramanien

Alison Kemp

Jaiganesh Mohan

**Warwick Hospital**

Ben Lee

Voon Kune Lim

Joshua Bower

**Watford General Hospital**

Mark Fullard

Paul Wolfson

**West Middlesex University Hospital**

Georgina Chadwick

George Hiner

**Western Isles Hospital**

Fraser Brooks

**Whipps Cross University Hospital**

Sami Hoque

Danujan Sriranganathan

**Whiston Hospital**

Vanessa Theis

Vanessa Theis

Cynthia Srikanathan

Emma Berryman

David McClements

Emily Wooley

**Wrexham Maelor Hospital**

Duncan Stewart

Will Thompson

Jack Barrington

Kasthuri Nallathamby

Samuel Thomas

Shaarven Kumar Jayachanra Moorthy

**Wycombe Hospital**

David Gorard

Mohamed Ibrahim

**Wythenshawe Hospital**

Dipesh Vasant

Ayodele Sasegbon

**Yeovil Hospital**

Fatima Elamin

**Ysbyty Gwynedd**

Jonathan Sutton
